# Lymphoproliferation in inborn errors of immunity: From challenging diagnosis to histologic revision

**DOI:** 10.70962/jhi.20250174

**Published:** 2026-02-13

**Authors:** Mattia Moratti, Beatrice Rivalta, Antonello Cardoni, Veronica Santilli, Enrico Attardi, Emma Concetta Manno, Riccardo Ciudino, Silvia Di Cesare, Cristina Cifaldi, Chiara Mengoli, Edoardo Muratore, Samuele Naviglio, Paola Selva, Simona Ferrari, Gigliola Di Matteo, Alessandro Broccoli, Nicola Cotugno, Donato Amodio, Riccardo Masetti, Elena Facchini, Pier Luigi Zinzani, Marcello Lanari, Cinzia Milito, Alberto Tommasini, Rita De Vito, Andrea Finocchi, Rita Alaggio, Elena Sabattini, Caterina Cancrini, Francesca Conti

**Affiliations:** 1 Department of Biomedicine and Prevention, Molecular Medicine and Applied Biotechnology, University of Rome Tor Vergata, Rome, Italy; 2 Pediatric Unit, IRCCS Azienda Ospedaliero-Universitaria di Bologna, University of Bologna, Bologna, Italy; 3 Unit of Clinical Immunology and Vaccinology, IRCCS Bambino Gesù Children Hospital, Rome, Italy; 4 Pathology Unit, Bambino Gesù Children’s Hospital, IRCCS, Rome, Italy; 5 Department of Biomedicine and Prevention, University of Rome Tor Vergata, Rome, Italy; 6 Haematopathology Unit, IRCCS Azienda Ospedaliero-Universitaria di Bologna, Bologna, Italy; 7 Department of Systems Medicine, University of Rome Tor Vergata, Rome, Italy; 8 Pediatric Hematology and Oncology, IRCCS Azienda Ospedaliero-Universitaria di Bologna, Bologna, Italy; 9 Department of Pediatrics, Institute for Maternal and Child Health IRCCS Burlo Garofolo, Trieste, Italy; 10 Laboratory of Immuno-Haematology-Laboratorio Unico Metropolitano, Azienda USL, Bologna, Italy; 11 Medical Genetics Unit, IRCCS Azienda Ospedaliero-Universitaria di Bologna, Bologna, Italy; 12 Research Unit of Primary Immunodeficiency, IRCCS Bambino Gesù Children Hospital, Rome, Italy; 13 IRCCS Azienda Ospedaliero-Universitaria di Bologna, Istituto di Ematologia “Seràgnoli,” Bologna, Italy; 14 Department of Medical and Surgical Sciences, University of Bologna, Bologna, Italy; 15 Department of Molecular Medicine, “Sapienza” University of Rome, Rome, Italy; 16 Department of Medical, Surgical and Health Sciences, University of Trieste, Trieste, Italy; 17 Department of Medical-Surgical Biotechnological Sciences, Sapienza University of Rome, Rome, Italy

## Abstract

Inborn errors of immunity (IEI) are genetic disorders that not only heighten infection risk but also disrupt immune regulation, frequently leading to lymphoid tissue overgrowth known as lymphoid proliferations (LPD). We retrospectively reviewed 38 patients with genetically or clinically confirmed IEI and persistent LPD, comparing those with nonneoplastic/reactive hyperplasia to those who developed overt lymphoid neoplasm (lymphoma). Overall, 26% developed lymphoma—predominantly classical Hodgkin lymphoma or diffuse large B cell lymphoma—often after earlier IEI onset. Immunophenotyping and principal component analysis revealed that patients with common variable immunodeficiency developing Hodgkin lymphoma shared a distinctive T cell profile, differing from immunocompetent lymphoma cases. Centralized histologic re-evaluation reclassified several presumed lymphoma as nonneoplastic/reactive hyperplasia and identified Castleman-like and germinal center transformation patterns in nonneoplastic/reactive LPD. Notably, elevated blood IgM and circulating T follicular helper cells mirrored IgM deposits and PD-1^+^ T cells in lymph nodes. These findings highlight the importance of an integrated approach involving clinical, genetic, and pathological reviews to improve IEI diagnosis and avoid overtreatment.

## Teaser

Moratti et al. exemplify how an effective management of IEI-related LPD demands close collaboration among immunologists, hematologists, and pathologists—integrating clinical presentation with serology, histology, immunohistochemistry, clonality assays, EBV status, and genetics. Reliance on histopathology alone risks misclassification, and atypical cases should prompt re-biopsy and multidisciplinary genetic reassessment to avoid overtreatment.

## Introduction

Inborn errors of immunity (IEI) constitute a growing and heterogeneous group of hereditary disorders affecting immune system development, regulation, and function (1, 2). These conditions demonstrate substantial variability in clinical expression—even among individuals with the same genetic mutation. While historically viewed as primary immunodeficiencies characterized by increased susceptibility to infections, IEI are now recognized as a spectrum of disease encompassing both infectious vulnerability and immune dysregulation (3, 4, 5). The latter includes autoimmunity, allergy, hyperinflammation, and lymphoid proliferations (LPD), which span a spectrum from nonneoplastic/reactive proliferations, through polymorphic immunodeficiency-associated LPD, to overt lymphoid neoplasms (lymphomas), in accordance with the World Health Organization (WHO) classification of hematolymphoid tumors (2024) (WHO-HAEM5) (6).

To assist in the classification of these complex disorders, the International Union of Immunological Societies (IUIS) has grouped IEI into 10 major categories based on shared clinical and immunological phenotypes (1, 2). Reflecting the growing recognition of LPD in the context of IEI, the WHO-HAEM5 (2024) introduced a dedicated section for LPD arising in individuals with IEI (6). These lesions range from nonneoplastic/reactive hyperplasia to polymorphic immunodeficiency-associated LPD and to overt lymphoid neoplasms (lymphomas) as defined by WHO-HAEM5. Sometimes distinguishing between polymorphic immunodeficiency-associated LPD and overt lymphomas poses significant diagnostic challenges due to some overlapping histological features, with important therapeutic implications (3, 6, 7, 8).

Moreover, in patients presenting with overt lymphoid neoplasms (lymphomas), an underlying IEI may remain undiagnosed. Awareness of red flags such as persistent or recurrent lymphoproliferation, atypical infections, chronic Epstein–Barr virus (EBV) replication, autoimmune phenomena, or syndromic features can help hemato-oncologists and pathologists to suspect an underlying IEI (9). Early genetic diagnosis of IEI has important therapeutic implications, potentially enabling individualized treatment strategies, improved disease control, and timely consideration for hematopoietic stem cell transplantation (HSCT) (7). In some IEI, targeted therapies are already available, allowing control of immune dysregulation and improved HSCT outcomes—particularly when performed at a younger age and in patients with a lower immune dysregulation index (10). However, prognostic tools remain limited in many IEI, and reliable biomarkers to predict disease evolution are still lacking (11).

Importantly, lymphoma risk represents a major determinant of reduced quality-adjusted life expectancy in patients with IEI (12). Therefore, predictive models to assess the lymphoma risk in patients with an LPD could therefore greatly improve patient care.

This study aimed to retrospectively analyze the histologic and immunologic features of IEI patients with LPD, comparing those with overt lymphoid neoplasm (lymphoma) versus nonneoplastic/reactive forms. The goal was to identify clinical and histological markers that could alert clinicians and pathologists to consider IEI, facilitate the diagnosis of IEI-related LPD, and guide the selection of the most appropriate treatment strategy.

Our results highlight that the effective management of IEI-associated and complex LPD requires close collaboration between immunologists, hematologists, and pathologists; indeed, relying solely on histopathological analysis may lead to misclassification, particularly in atypical cases, which should prompt repeat biopsy and multidisciplinary genetic re-evaluation to prevent unnecessary treatment.

## Results

### General cohort features

A total of 85 patients with LPD were provisionally included in the study. Among them, 38 patients (45%) were subsequently diagnosed with an IEI on a clinical and/or genetic basis and were therefore definitively enrolled in the study (Table 1).

**Table 1.** Clinical and genetic features of the cohort

Patient/Gender	Final diagnosis	NGS panel/WES	Gene/variant/validation	Infections	Chronic EBV viremia	Nonneoplastic/reactive LPD	Overt lymphoma	Autoimmunity	Other immune dysregulation	Treatment for immune dysregulation	IRT	Outcome	Follow-up
P1/M	CID	WES (not informative)	/	Pneumonia (17 years) RRI	Yes (17 years)	Hepatosplenomegaly and laterocervical, supraclavicular and inguinal lymphadenopathies (13 years)	/	/	Nephrotic syndrome (2.5 years)	Steroids, rituximab, MMF	ScIg	Alive	10 years
P2/F	CID	WES (not informative)	/	RRI (4 years) Pneumonia (6 years)	/	Hepatosplenomegaly, laterocervical and mediastinal lymphadenopathy, and GLILD (8 years)	/	/	Atopic dermatitis	HD-IvIg, rituximab, MMF	IvIg	Alive	7 years
P3/M	CID	WES (not informative)	/	RRI (6 years) Atypical mycobacterial infection	/	Hepatosplenomegaly, peri-hilar hepatic and peri-encephalo-pancreatic lymphadenopathy, and GLILD (9 years)	/	ITP (6 years) AIHA	/	Steroids, rituximab, rapamycin	ScIg	Alive	6 years
P4/M	CID	WES (not informative)	PIK3CD Heterozygous c.550C>T_p.(Arg184Trp) (VUS)	RRI (0.5 year)	/	Splenomegaly and diffuse lymphadenopathy (2 years)	/	ITP and AIHA (2 years)	IBD Chronic arthritis	HD-IvIg, MMF	IvIg	Deceased upon allogenic HSCT	/
P5/F	CID	WES (not informative)	/	Chronic CMV and EBV infections with colitis and chronic hepato-pancreatitis (0.3 year)	Yes (0.3 year)	Splenomegaly, inguinal and mandibular lymphadenopathy (0.8 year)	/	/	IBD	HD-IvIg, rituximab	IvIg	Alive	16.5 years
P6/M	CID	NGS IEI panel^a^	LRBA Heterozygous c.2949G>T_p.(Cys950Phe) (VUS)	HBV-related cirrhosis	Yes (53 years)	Submandibular, laterocervical, subclavian, axillar, and iliac lymphadenopathy (50 years)	/	/	Asthma	Rituximab	/	Deceased upon *P. aeruginosa* sepsis	/
P7/F	CID	NGS IEI panel (ongoing)^a^	/	HPV cervical and tonsillar lesions (44 years)	/	Subclavian, retroscapular, mediastinal, abdominal, and retroperitoneal lymphadenopathy (53 years)	/	/	Recurrent periarthritis and tendonitis	/	/	Alive	1 year
P8/M	CID-like	WES	PIK3CD Heterozygous c.935C>G_p.(Ser312Cys) (benign)	RRI and pneumonia (0.5 year) *P. aeruginosa* pneumonia (3 years) Salmonellosis (3 years)	/	Splenomegaly, pulmonary, and mediastinal lymphadenopathy (3 years)	*/*	/	/	/	/	Alive	7 years
**P9/F**	**ATM**	**NGS IEI panel** ^a^	***ATM*** **Compound heterozygous c.2413C>T_p.(Arg805Ter) (known pathogenic)** **c. 6996_6999del_p.(Thr2333fsX5**) **(known pathogenic/likely pathogenic)**	**RRI and urinary tract infections (2 years)** **HHV6 chronic viremia**	**/**	**Splenomegaly and diffuse lymphadenopathy (6 years)**	**DLBCL (10 years)**	**/**	**Primary sclerosing cholangitis**	**/**	**/**	**Deceased upon MOF triggered by sepsis**	**/**
**P10/M**	**ATM**	**NGS IEI panel** ^a^	***ATM*** **Compound heterozygous c.7517_7520del_p.(Arg2506ThrfsTer3)? (known pathogenic)** **c.5692C>T_ p.(Arg1898Ter) (known pathogenic)**	**RRI and pneumonia (3 years)**	**Yes (3 years)**	**Diffuse lymphadenopathy (5 years)**	**cHL (6 years)**	**/**	**Eczematous-granulomatous skin lesions**	**/**	**IvIg**	**Deceased upon MOF due to norovirus infection**	**/**
**P11/F**	**ATM**	**NGS IEI panel** ^a^	***ATM*** **Compound heterozygous c.2251_2376del_p.? (splice junction loss)** **c.6453_6572del_p.? (splice junction loss)**	**RRI (1.5 years)**	**Yes (5 years)**	**Splenomegaly, laterocervical, supraclavicular, and axillar lymphadenopathy, GLILD (5 years)**	**/**	**/**	**Thyroid carcinoma, hepatic adenoma**	**/**	**/**	**Alive**	**12 years**
P12/M (13, 14)	CVID	NGS IEI panel (not informative)^a^	*/*	*/*	*/*	Axillar, cervical and supraclavicular lymphadenopathy, and hepatosplenomegaly (23 years)	*/*	ITP and thyroiditis	Celiac disease	HD-IvIg, steroids	ScIg	Alive	4 years
P13/F (14)	CVID	NGS IEI panel (not informative)^a^	*/*	*/*	Yes	Splenomegaly, laterocervical, axillar, retropectoral, supradiaphragmatic and abdominal lymphadenopathy, and GLILD (57 years)	*/*	Hashimoto thyroiditis (30 years) ITP	Psoriasis and gastrointestinal inflammatory disorder	MMF	ScIg	Alive	9 years
P14/F (14)	CVID	NGS IEI panel (ongoing)^a^	*/*	RRI	Yes	Hepatosplenomegaly, retronucal, laterocervical, subclavian, axillar, para-aortic, iliac, inguinal, and femoral lymphadenopathy (18 years)	*/*	ITP (35 years)	Psoriasis	/	ScIg	Alive	5 years
P15/M (13)	CVID	NGS IEI panel (not informative)^a^	*/*	/	/	Submandibular, laterocervical, and axillar lymphadenopathy (14 years)	*/*	ITP (14 years)	Atopic dermatitis	HD-IvIg, steroids, MMF	IvIg	Alive	3 years
P16/F	CVID	NGS IEI panel (not informative)^a^	*/*	/	/	Subparotideal, jugulodigastric, submandibular, laterocervical, and inguinal lymphadenopathy (7 years)	Borderline NLPHL (7 years)	*/*	*/*	*/*	*/*	Alive	4 years
P17/F	CVID	NGS IEI panel (not informative)^a^	*/*	RRI and pneumonia (3 years) HSV meningitis	/	Splenomegaly, mesenteric and para-aortic lymphadenopathy (49 years)	*/*	*/*	*/*	*/*	ScIg	Alive	22 years
P18/M (13, 14, 15)	CVID	NGS IEI panel (not informative)^a^	*/*	Recurrent bronchopneumonia (34 years)	/	Splenomegaly, mediastinal and inter-aorto-caval lymphadenopathy (17 years)	/	ITP (17 years)	Reactive arthritis	HD-IvIg, steroids, rituximab	ScIg	Alive	3 years
**P19/M**	**CVID**	**NGS IEI panel** ^a^	***TNFRSF13B* (*TACI*)** **Heterozygous c.310T>C_p.(Cys104Arg) (known pathogenic)**	**Visceral leishmaniasis (27 years)** **Warts**	**/**	**Splenomegaly and gastrointestinal lymphoid hyperplasia (27 years)**	**/**	**AIN and ITP (2 years)**	**Atopic dermatitis**	**Steroids, rituximab, rapamycin**	***/***	**Alive**	**2 years**
P20/M (13, 14)	CVID	NGS IEI panel^a^	*CR2* Heterozygous c.3074C>T_p.(Ala1025Val) (VUS)	RRI and pneumonia (28 years) Chronic CMV viremia HPV high-grade skin lesions	/	Splenomegaly, pulmonary hilar and mediastinal lymphadenopathy, and GLILD (30 years)	/	ITP (25 years)	/	Rituximab, MMF	ScIg	Alive	6 years
P21/M (13, 14, 15)	CVID	NGS IEI panel (not informative)^a^	*/*	RRI and urinary tract infections (2 years) Chronic CMV viremia and pneumonia*Branhamella* * catarrhalis* pneumonia	Yes	Hepatosplenomegaly, axillar, hilar-mediastinal, inter-aorto-caval, para-aortic, intra- and retro-peritoneal lymphadenopathy, and GLILD (10 years)	/	ITP and AIHA (10 years)	/	HD-IvIg, steroids, MMF	ScIg	Alive	6 years
P22/M (16)	CVID	WES	*TNFRSF13C* Heterozygous c.475C>T_p.(His159Tyr) (conflicting interpretations of pathogenicity; reduced BAFFR expression on B cells and T follicular helper and regulatory cells)	Pulmonary atypical mycobacterial infection (20 years)	/	Hepatosplenomegaly, laterocervical, subclavian, mediastinal, iliac, peri-hepatic, abdominal, and pelvic lymphadenopathy (20 years)	/	ITP and DM1 (16 years)	Asymmetric axonal sensory polyneuropathy (18 years)	HD-IvIg, steroids, rituximab	ScIg	Alive	5 years
P23/M	CVID	WES (not informative)	*/*	RRI (8 years) Atypical mycobacterial infection EBV pneumonia	/	Hepatosplenomegaly, axillar, hilar mediastinal, abdominal, and pelvic lymphadenopathy, and GLILD (16.5 years)	/	/	/	Rituximab	IvIg	Alive	3.5 years
P24/M	CVID	NGS IEI panel^a^	*PIK3R1* Heterozygous c.-208_-204del_p.? (likely benign [trio-WES ongoing])	Dental abscess (3 years)	Yes (5 years)	Hepatosplenomegaly, laterocervical and subclavian lymphadenopathy (5 years)	cHL (5 years) cHL (7 years) cHL (8 years)	/	/	/	IvIg	Alive	2 years
P25/M	CVID-like	WES	*SLC7A7* Heterozygous c.1370dupA_p.(Tyr457Ter) (likely pathogenic) *SBDS* Heterozygous c.258+2T>C_p.(Cys84fsTer3) (pathogenic) *CR2* Heterozygous c.1676G>A_(p.Gly559Glu) (VUS)	/	/	Hepatosplenomegaly, submandibular, laterocervical, and axillar lymphadenopathy (4 years)	DLBCL (14 years)	/	Atopic dermatitis	/	ScIg	Alive	6.5 years
P26/F	CVID-like^c^	WES	*/*	RRI, pneumonia Chronic CMV viremia	Yes	Splenomegaly, submandibular and laterocervical lymphadenopathy (8.5 years)	BL (5 years)	/	/	/	ScIg	Alive	7 years
**P27/F** (17, 18, 19)	**APDS2**	**NGS IEI panel** ^a^	***PIK3R1*** **Heterozygous c.1300-2A>G_p.? (exon 11 complete skipping at cDNA analysis)**	**RRI and pneumonia (14 years)**	**/**	**Hepatosplenomegaly, axillar and hilar mediastinal lymphadenopathy (20 years)**	**/**	**SLE (ITP, AIHA) (20 years)**	**Chilblains, arthritis, cardiomyopathy, serositis**	**Steroids, rapamycin, PI3Kδ inhibitor**	**ScIg**	**Alive**	**2 years**
**P28/M** (20, 21)	**APDS1**	**WES**	***PIK3CD*** **Heterozygous c.3061G>A_p.(Glu1021Lys) (known pathogenic)**	**RRI (0.6 year)** **Atypical mycobacterial infection**	**Yes**	**Hepatosplenomegaly, diffuse lymphadenopathy (0.6 year)**	**DLBCL (20 years)** **DLBCL (24 years) DLBCL/cHL (26 years)**	**AHIA, hepatitis**	**Pericarditis**	**HD-IvIg, steroids, rituximab, PI3Kδ inhibitor**	**IvIg**	**Alive**	**22 years**
**P29/F** (20)	**APDS2**	**WES**	***PIK3R1*** **Heterozygous c.1425+1G>T_p.? (known pathogenic)**	**RRI (0.7 year)**	**Yes (17 years)**	**Hepatosplenomegaly, tonsillar laterocervical, axillar, and inguinal lymphadenopathy (5 years)**	**cHL (18 years)** **DLBCL (30 years)** **cHL (37 years)**	**Thyroiditis**	**IBD**	**Steroids, rituximab**	**ScIg**	**Alive**	**26 years**
**P30/F**	**APDS1**	**WES**	***PIK3CD*** **Heterozygous c.3061G>A_p.(Glu1021Lys) (known pathogenic)**	**RRI and pneumonia (0.5 year)**	**Yes**	**Hepatosplenomegaly, diffuse lymphadenopathy (4 years)**	**/**	**/**	**IBD and vernal keratoconjunctivitis**	**/**	**/**	**Alive**	**1 year**
**P31/F** (20, 22, 23)	**APDS2**	**NGS IEI panel** ^b^	***PIK3R1*** **Heterozygous c.1425+1G>T_p.? (known pathogenic)**	**RRI and pneumonia (0.7 year)**	**Yes (3 years)**	**Splenomegaly, tonsillar, submandibular, and laterocervical lymphadenopathy (2 years)**	**/**	**/**	**/**	**Steroids, rapamycin, theophylline, PI3Kδ inhibitor**	**ScIg**	**Alive**	**13 years**
**P32/F**	**CTLA4 deficiency**	**NGS IEI panel** ^a^	***CTLA4*** **Heterozygous c.394G>A_p.(Glu132Lys) (impaired CD80 transendocytosis)**	**RRI** **VZV reactivation** **Sepsis** **Paravertebral abscess** **Warts** **CMV chronic viremia**	**/**	**Splenomegaly, mediastinal and retroperitoneal lymphadenopathy (15 years)**	**/**	**Thyroiditis (15 years)**	**Central nervous system and pulmonary inflammation and granulomatous disorder (15 years)**	**HD-IvIg, steroids, abatacept**	**ScIg**	**Alive**	**1 year**
**P33/F** (24)	**CTLA4 deficiency**	**NGS IEI panel** ^b^	***CTLA4*** **Heterozygous c.223C>T_p.(Arg75Trp) (known pathogenic)**	**/**	**/**	**Splenomegaly, lymphadenopathy, GLILD (15 years)**	**/**	**ITP, AIHA, thyroiditis (15 years)**	**Psoriasis**	**/**	**ScIg**	**Alive**	**5 years**
**P34/M** (13)	**ALPS**	**NGS IEI panel** ^a^	***TNFRSF6* (*FAS*)** **Heterozygous c.856G>T_p.(Gly286X) (defective FAS-induced lymphocyte apoptosis assay)**	**/**	**/**	**Splenomegaly, lymphadenopathy (8 years)**	**/**	**AIHA (8 years)**	**/**	**HD-IvIg, steroids, rituximab, MMF**	**ScIg**	**Alive**	**17 years**
**P35/M** (13)	**ALPS**	**NGS IEI pane**l^a^	***TNFRSF6* (*FAS*)** **Heterozygous c.568G>A_p.(Val190Met) (defective FAS-induced lymphocyte apoptosis assay)**	**/**	**Yes**	**Hepatosplenomegaly, laterocervical, axillar, abdominal, and inguinal lymphadenopathy (8 years)**	**/**	**/**	**/**	**/**	**/**	**Alive**	**13 years**
P36/M	ALPS-like	NGS IEI panel (ongoing)^a^	*/*	Recurrent otitis (2 years)	/	Laterocervical, axillar, and inguinal lymphadenopathy (14 years)	/	ITP (14 years)	Atopic dermatitis	Rapamycin	/	Alive	1 year
**P37/M**	**sALPS**	**WES**	***TNFRSF6* (*FAS*)** **Heterozygous c.778G>A_p.(Asp260Asn) (known pathogenic)**	**/**	**/**	**Hepatosplenomegaly, submandibular and laterocervical lymphadenopathy (5 years)**	**/**	**ITP and AIHA (5 years)**	**/**	**Rapamycin**	**/**	**Alive**	**1 year**
**P38/M**	**XMEN**	**WES**	***MAGT1*** **Hemizygous c.370C>T_p.(Gln124Ter) (pathogenic)**	**/**	**Yes (5 years)**	**Retroauricular and laterocervical lymphadenopathy (5 years)**	**cHL (5 years)**	**ITP (3.5 years)**	**/**	**Rituximab**	**IvIg**	**Alive**	**3 years**

Patients with a validated genetic IEI diagnosis are reported in bold. The age at onset of signs is indicated in parentheses for each variable included. AIN, autoimmune neutropenia; CMV, cytomegalovirus; BL, Burkitt lymphoma; DM1, type 1 diabetes mellitus; F, female; GLILD, granulomatous lymphocytic interstitial lung disease; HBV, human hepatitis B virus; HPV, human papilloma virus; HSV, herpes simplex virus; IBD, inflammatory bowel disease; IRT, immunoglobulin replacement therapy; ITP, immune thrombocytopenia; M; male; MMF, mycophenolate mofetil; MOF, multi-organ failure; RRI, recurrent respiratory infections; ScIg, subcutaneous immunoglobulins; SLE, systemic lupus erythematosus; VUS, variant of uncertain significance; VZV, Varicella Zoster virus; HHV-6, human herpesvirus 6; PI3Kδ, phosphoinositide 3-kinase delta.

a Patient tested using a 49-gene panel for IEI typically associated with immune dysregulation.

b Patient tested using a 525-gene panel for IEI.

c P26 was included in the CVID(-like) group, being admitted with a post-rituximab hypogammaglobulinemia.

**Figure 1.**
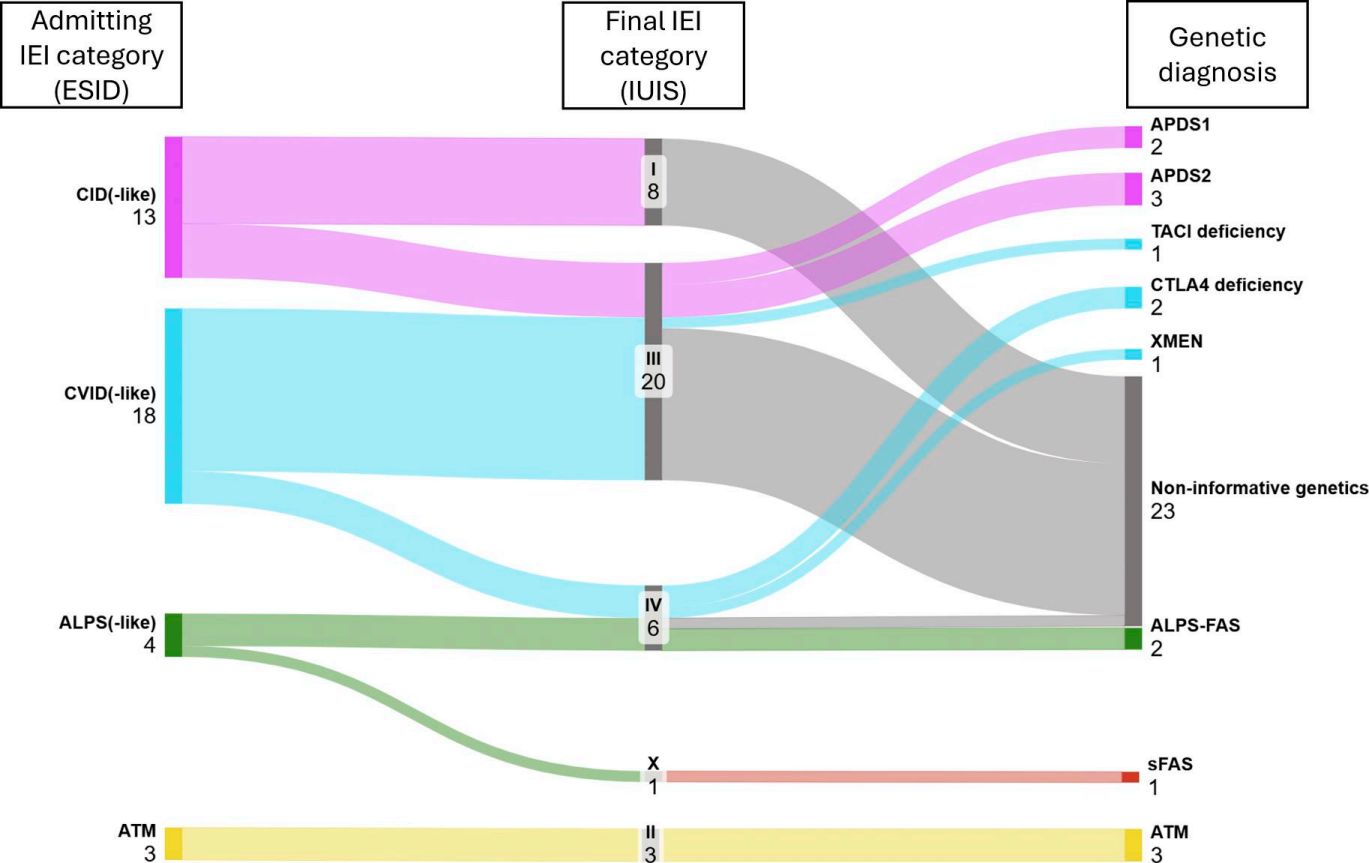
**Diagnostic flow from admitting to genetic IEI diagnosis.** Numbers refer to patients within each category. ESID, European Society for Immunodeficiencies

The most frequent admitting clinical–immunological diagnosis prior to genetic testing was common variable immunodeficiency (CVID) or CVID-like disorder, reported in 18 patients (47%). Within this group, genetic analysis identified one case of transmembrane activator and CAML interactor (TACI) haploinsufficiency, two cases of cytotoxic T lymphocyte-associated protein 4 (CTLA4) deficiency, and one case of X-linked immunodeficiency with magnesium defect (XMEN) syndrome. Combined immunodeficiency (CID)–like disorders represented the second most common admitting category, including 13 patients (34%)—among whom two were diagnosed with activated PI3K-δ syndrome (APDS) one APDS2, and three with APDS2. Autoimmune lymphoproliferative syndrome (ALPS) and ALPS-like presentations were observed in four patients (10%); genetic testing confirmed ALPS in two cases and an ALPS phenocopy with a pathogenic somatic *FAS* variant in one. Finally, three patients (8%) with clinical and laboratory suspicion of ataxia-telangiectasia (ATM) received a genetically confirmed diagnosis (Fig. 1).

### Histological revision

Among the 26/38 patients (68%) who underwent excisional biopsies, nine (35%) required multiple procedures during their clinical course, with an average of approximately three tissue biopsies per patient in this subgroup.

Histological re-examination was performed based on all clinical information retrospectively collected in 21 out of 25 patients (excluding P10, P25, P26, and P38). This was conducted on complete nodal excision in 20 of the 21 cases and on intestinal tissue in the remaining patient (P29), in accordance with the 2024 WHO-HAEM5 (6).

Lesions were categorized as (i) nonneoplastic/reactive LPD (including follicular hyperplasia, paracortical expansion, mixed hyperplasia, and Castleman-like patterns), (ii) polymorphic immunodeficiency–associated LPD, or (iii) overt lymphoid neoplasms (lymphomas). When histologic features were borderline, the final designation incorporated clinical, EBV, clonality, and immunophenotypic data; cases managed as lymphoma for therapeutic reasons are reported as overt lymphoma in analyses.

Histologic confirmation of nonneoplastic/reactive LPD was achieved in all 16 patients initially admitted with presumed reactive disease. Among these, four patients subsequently developed overt lymphoid neoplasms (lymphomas), confirmed upon histologic re-evaluation. In four out of 13 patients (31%) initially diagnosed with lymphoma, histological revision resulted in reclassification as nonneoplastic/reactive LPD, consistent with WHO-HAEM5 terminology. The revised cases included two instances of misdiagnosed late-onset splenic marginal zone lymphoma—one involving a misrecognized T cell lymphoid expansion with a modest component of medium-to-large B cells in a 49-year-old female patient with CVID (P17), and the other a misrecognized mixed hyperplasia with irregular polyclonal rearrangement of immunoglobulin (Ig) heavy and kappa light chains in a 37-year-old male patient with CVID (P18). Additionally, two initial diagnoses of classical Hodgkin lymphoma (cHL) were subsequently revised to nonneoplastic/reactive conditions: follicular hyperplasia with exuberant B cell activation in a 28-year-old male patient with TACI deficiency (P19) and paracortical expansion with exuberant B cell activation in a female patient with APDS2 (P27), treated at age 19 with intensive chemotherapy and autologous HSCT (17).

**Figure 2.**
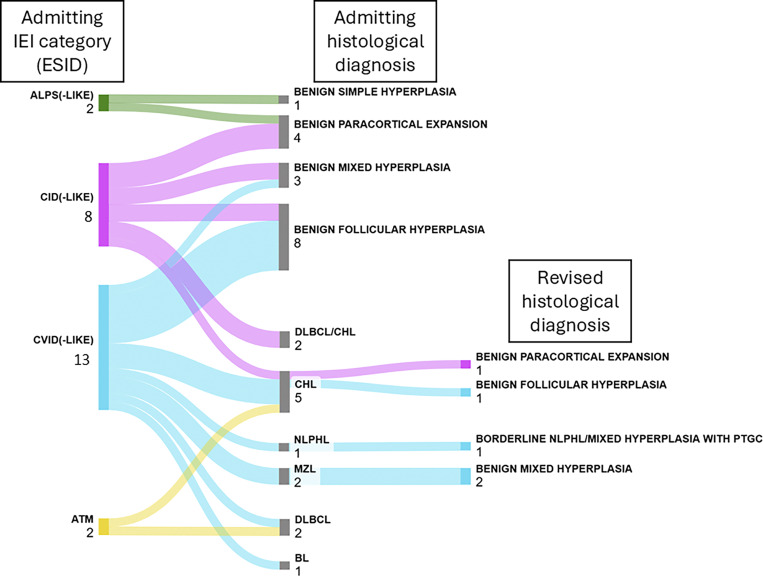
**Diagnostic flow from admitting to revised LPD histological diagnosis.** Numbers refer to patients within each category. BL, Burkitt lymphoma; ESID, European Society for Immunodeficiencies; MZL, marginal zone lymphoma.

It is worth mentioning that P16 (CVID originally diagnosed with lymphoma) exhibited a borderline LPD between nodular lymphocyte predominant HL (NLPHL) and mixed hyperplasia with a progressive transformation of germinal center (PTGC)–like pattern. Given the persistent diagnostic uncertainty upon histologic reassessment, the case was managed as lymphoma and included in the group of patients with overt lymphoid neoplasm (lymphoma) (Fig. 2).

Among the 20 patients with confirmed nonneoplastic/reactive LPD for whom excisional lymph node biopsies were performed, 12 (60%) had a not–genetically defined CVID or CVID-like condition, three (15%) had a not-genetically defined CID(-like), three (15%) were diagnosed with APDS, one with an ALPS-like condition, and one with somatic ALPS (sALPS).

**Figure S1.**
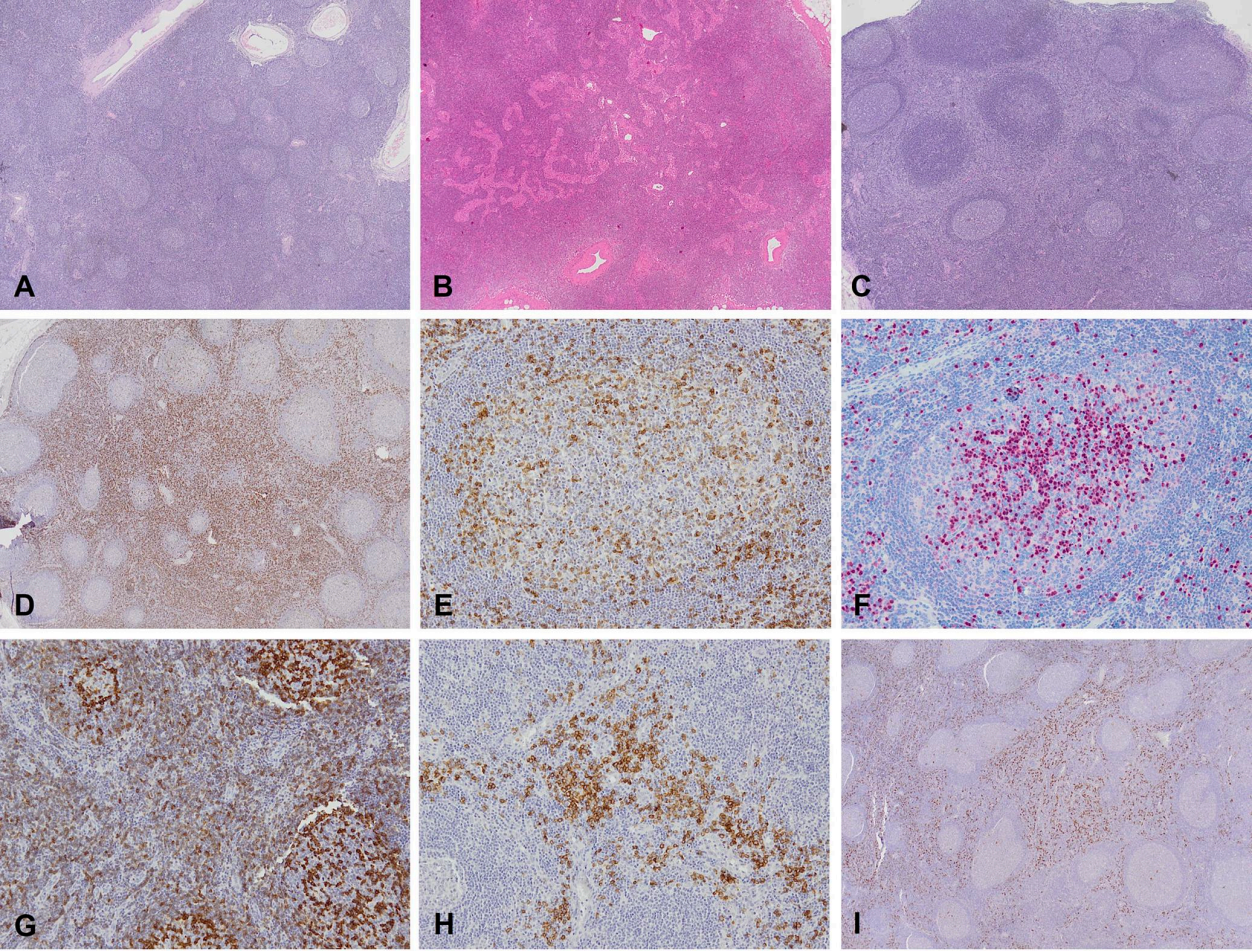
**Histopathologic findings. (A–I)** Follicular hyperplasia (2X, H&E); (B) paracortical expansion (2X, H&E); (C) PTGC-like features (2X, H&E); (D) CD8^+^ T cells (2X); (E) CD8^+^ T cells in GC (10X); (F) IRF4+ plasma cells in GC (10X); (G) PD1+ T cells (10X); (H) pDCs CD163+ (10X); (I) pDCs CD303+ (2X). H&E, hematoxylin and eosin.

**Figure S2.**
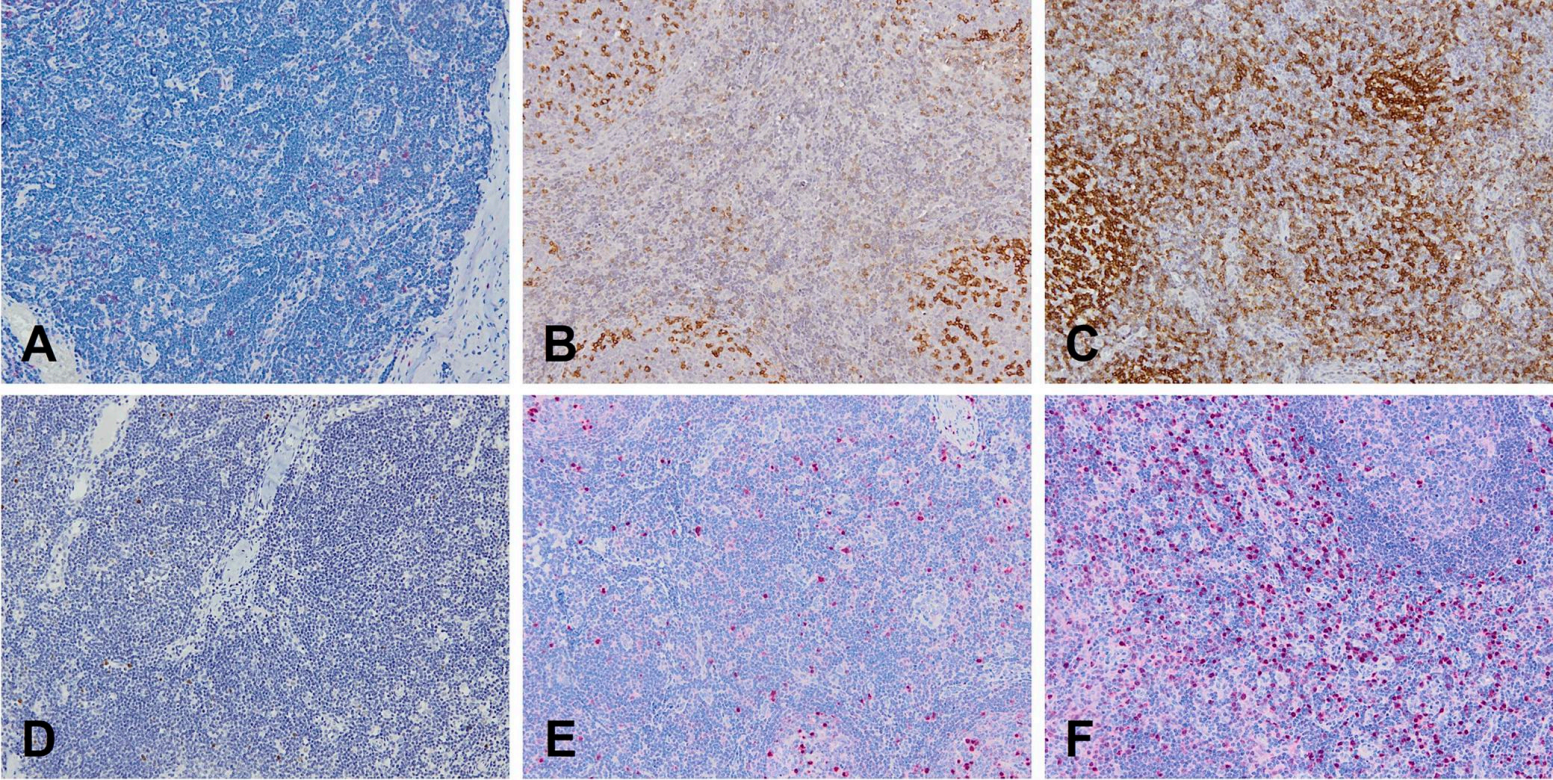
**Representative examples of tissue plasma cells and interfollicular PD1-positive T cells quantification. (A–F)**: (A–C): PD1+ T cells (10X); (D–F): IRF4+ interfollicular plasma cells (10X). A and D correspond to <Q1, B and E to Q1–Q3, and C and F to >Q3 in heatmap (Table 2).

These nonneoplastic/reactive LPD underwent an in-depth histological analysis, reported in Table 2. Representative images of the main histopathological findings and of histologic features are reported in Figs. S1 and S2.

**Table 2.** Histologic features of nonneoplastic/reactive lymph nodal LPD (frequency and heatmap)

Histologic and immunologic features	Not**–**genetically defined CID(-like)				CVID/CVID-like								APDS2	APDS1	ALPS-like	sALPS	Features frequency
	P1	P2	P8	P9	P12	P13	P14	P15	P16	P22	P25	P26	P27	P28	P36	P37	
Follicular B hyperplasia	−	−	−	−	+	+	+	+	−	+	+	+	−	+	−	−	50%
Paracortical T hyperplasia	+	+	−	+	−	−	−	−	−	−	−	−	+	−	−	+	31%
Mixed T/B hyperplasia	−	−	+	−	−	−	−	−	+	−	−-	−	−	−	−	−	12%
Simple hyperplasia	−	−	−	−	−	−	−	−	−	−	−	−	−	−	+	−	6%
Castleman-like features	+	NA	−	−	−	−	−	−	−	−	−	−	−	−	−	+	12%
PTGC-like features	−	NA	−	−	−	−	−	−	+	−	+	−	−	−	−	−	12%
Granulomas	−	+	−	−	+	−	−	−	−	−	−	−	−	−	−	−	12%
CD8^+^ T cells increase in GC	−	−	+	+	−	−	−	+	+	−	−	−	−	−	−	−	25%
TIA1 increase in comparison with CD8^+^ T cells	+	−	−	−	−	−	−	−	−	−	−	−	−	+	−	−	12%
Focal EBER expression increase in a single GC with EBV reactivation signs	+	−	−	+	−	−	NA	NA	−	−	−	−	−	−	−	−	12%
Chronic EBV replication with viremia >1,000 copies/ml	+	−	+	−	−	+	+	−	−	−	−	+	−	+	−	−	37%
Increased GC PD1+ follicular helper T cells	+	−	−	−	−	+	−	−	−	+	+	−	−	+	−	−	31%
Blood CXCR5+CD45RA−CD4+ Tfh cells increase	+	−	NA	NA	NA	NA	NA	NA	NA	​	−	−	NA	+	NA	−	43%
CD138+ plasma cells increase in GC	−	NA	NA	NA	NA	NA	NA	NA	NA	−	−	−	NA	−	NA	−	0%
IRF4+ plasma cells increase in GC	−	−	−	−	NA	−	+	+	+	−	−	−	−	−	−	−	19%
Blood plasmablasts increase	−	−	−	−	NA	−	−	−	+	+	−	−	+	NA	−	+	25%
IgG4 increase in GC	−	−	−	−	−	−	−	−	−	−	−	−	−	−	+	−	6%
Interfollicular CD68+/CD163+ macrophages increase	+	NA	+	−	−	+	−	−	−	−	−	−	+	−	−	−	25%
CD303+/CD123+ pDCs aggregates (>3)	+	​	+	+	NA	+	+	−	+	+	+	−	−	−	+	+	62%
CD30+ immunoblasts increase	−	−	+	−	−	−	−	−	+	−	−	−	−	+	−	−	19%
Interfollicular CD4/CD8 ratio skewed toward CD8+ T cells	Q1–Q3	NA	Q1–Q3	>Q3	Q1–Q3	<Q1	Q1–Q3	Q1–Q3	Q1–Q3	Q1–Q3	Q1–Q3	NA	Q1–Q3	Q1–Q3	Q1–Q3	Q1–Q3	​
Interfollicular PD1 expression in exhausted T cells	<Q1	<Q1	>Q3	>Q3	Q1–Q3	Q1–Q3	>Q3	Q1–Q3	Q1–Q3	Q1–Q3	<Q1	<Q1	>Q3	Q1–Q3	Q1–Q3	<Q1	​
Interfollicular IRF4+ plasma cells (3 HPF:40× magnification)	Q1–Q3	<Q1	Q1–Q3	<Q1	Q1–Q3	<Q1	Q1–Q3	<Q1	>Q3	>Q3	Q1–Q3	Q1–Q3	Q1–Q3	Q1–Q3	>Q3	>Q3	​
Interfollicular CD138+ plasma cells (3 HPF:40× magnification)	Q1–Q3	NA	NA	<Q1	NA	NA	NA	<Q1	Q1–Q3	>Q3	NA	NA	NA	NA	>Q3	NA	​
Interfollicular IgM count (3 HPF:40× magnification)	>Q3	Q1–Q3	<Q1	Q1–Q3	Q1–Q3	<Q1	Q1–Q3	Q1–Q3	<Q1	Q1–Q3	<Q1	<Q1	>Q3	>Q3	<Q1	Q1–Q3	​
GC IgM count (3 HPF:40× magnification)	>Q3	NA	>Q3	Q1–Q3	Q1–Q3	Q1–Q3	Q1–Q3	Q1–Q3	Q1–Q3	<Q1	Q1–Q3	Q1–Q3	<Q1	>Q3	Q1–Q3	<Q1	​
Serum IgM	>Q3	<Q1	<Q1	<Q1	<Q1	<Q1	<Q1	<Q1	Q1–Q3	Q1–Q3	Q1–Q3	Q1–Q3	>Q3	>Q3	Q1–Q3	<Q1	​
Interfollicular IgG count (3 HPF:40× magnification)	<Q1	<Q1	Q1–Q3	Q1–Q3	>Q3	<Q1	>Q3	<Q1	Q1–Q3	>Q3	Q1–Q3	Q1–Q3	Q1–Q3	Q1–Q3	Q1–Q3	>Q3	​
GC IgG count (3 HPF:40× magnification)	<Q1	<Q1	>Q3	Q1–Q3	Q1–Q3	Q1–Q3	>Q3	<Q1	<Q1	Q1–Q3	>Q3	Q1–Q3	NA	Q1–Q3	Q1–Q3	>Q3	​
Serum IgG	<Q1	Q1–Q3	<Q1	<Q1	<Q1	<Q1	<Q1	<Q1	Q1–Q3	>Q3	Q1–Q3	Q1–Q3	Q1–Q3	Q1–Q3	Q1–Q3	<Q1	​
Interfollicular IgD count (3 HPF:40× magnification)	Q1–Q3	Q1–Q3	Q1–Q3	Q1–Q3	Q1–Q3	Q1–Q3	Q1–Q3	>Q3	Q1–Q3	Q1–Q3	Q1–Q3	>Q3	Q1–Q3	Q1–Q3	>Q3	Q1–Q3	​
GC IgD count (3 HPF:40× magnification)	>Q3	Q1–Q3	Q1–Q3	Q1–Q3	Q1–Q3	Q1–Q3	Q1–Q3	>Q3	Q1–Q3	Q1–Q3	Q1–Q3	Q1–Q3	Q1–Q3	Q1–Q3	>Q3	Q1–Q3	​

In the first part of the table, presence of a variable is indicated by +, absence by −. In the bottom half of the table, each variable considered is represented by a scale of quartiles, in an increasing order of magnitude. Regarding the interfollicular and GC Ig count, <Q1 indicates values below the first quartile, Q1–Q3 is used for values between the first and third quartiles, and >Q3 represents values above the third quartile. NA, not assessed; PD1, programmed cell death protein 1.

**Figure S3.**
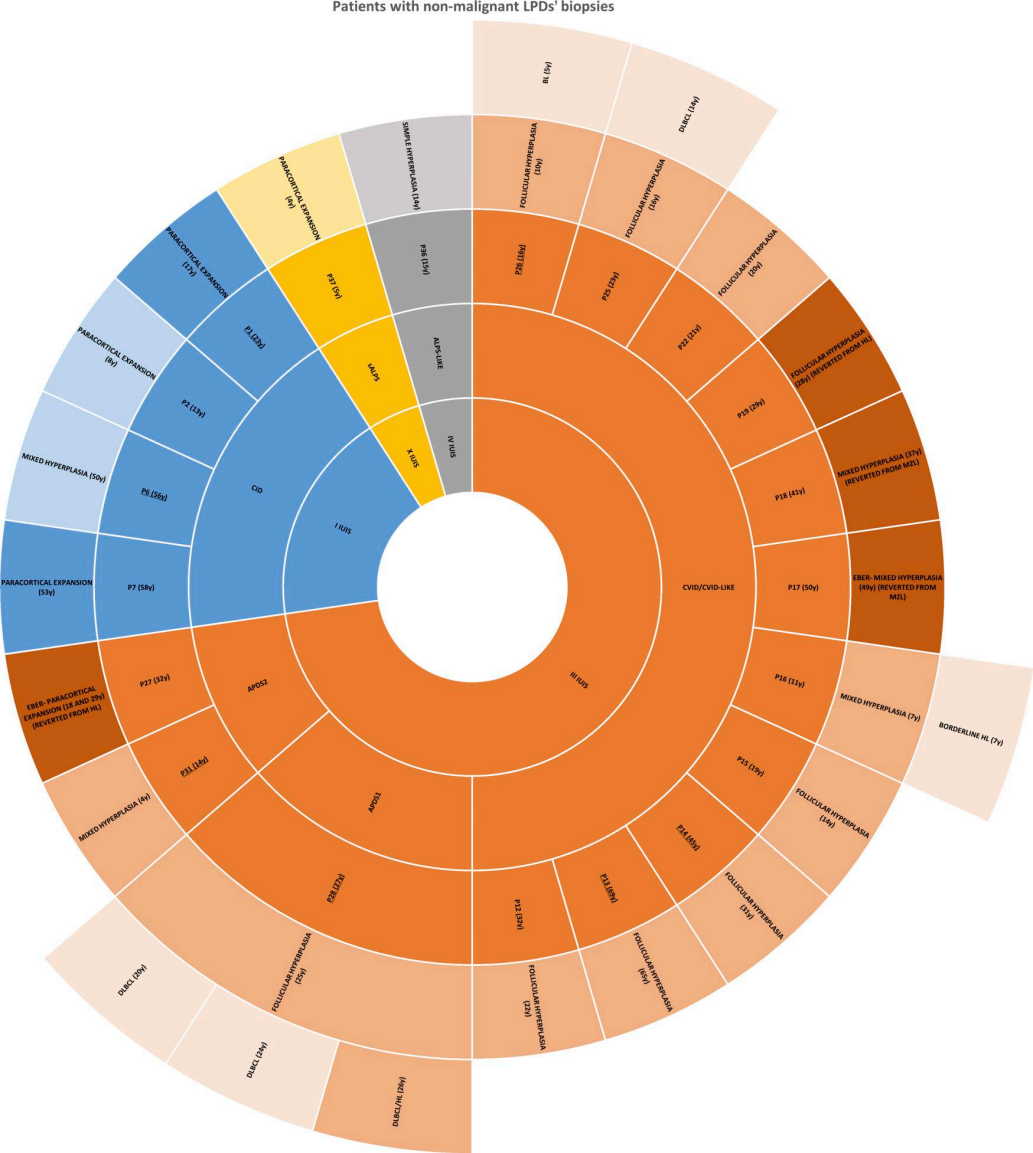
**Patients with nonneoplastic/reactive LPD biopsies.** The innermost ring categorizes patients based on the IUIS classification of IEI, showing that most nonneoplastic/reactive LPD requiring histological examination belong to category III (predominantly antibody deficiency). The second inner ring details specific IEI diagnoses. The third inner ring shows the identification number of each patient, reporting in parentheses the age at last follow-up; double-underlined patients are those experiencing EBV chronic replication with a viremia >1,000 copies/ml during follow-up. The fourth inner ring specifies the overall histologic features of nonneoplastic/reactive LPD, reporting in parentheses the age at biopsy; EBER-negative LPD are displayed in a lighter shade, distinguishing them from EBER-positive LPD, which retain the same color shade as their corresponding internal classification rings (except for P14, P15, P18, P19, and P31, for which EBER expression was not available). A darker shade is reserved to reverted diagnosis upon histologic reassessment. The outermost ring reports the type of lymphoma developed by the corresponding patient of the third inner ring; the age at lymphoma biopsy is reported in parentheses. EBER-negative lymphomas are displayed in a lighter shade, distinguishing them from EBER-positive lymphomas, which retain the same color shade as their corresponding internal classification rings.

The overall histologic features of nonneoplastic/reactive lymph node LPD can be grouped into four main categories, namely follicular hyperplasia (9/20 patients, 45%), paracortical T hyperplasia (5/20 patients, 2%), mixed B and T hyperplasia (5/20 patients, 25%), and simple hyperplasia in a single case. Finally, diagnosed CVID/CVID-like patients presented with follicular hyperplasia in 8/11 cases (Fig. S3).

In P1 and P37, with a non-genetically confirmed CID and sALPS diagnosis, respectively, we found Castleman-like features in the context of paracortical T hyperplasia in both cases. Interestingly, in P1, this histologic pattern was associated with an increase in germinal center (GC) PD1+ follicular helper T (Tfh) cells, corresponding to a vertiginous expansion in peripheral blood CXCR5+CD45RA−CD4+ Tfh (45.4% of CD4^+^CD45RA cells)—although blood sampling and excisional lymph node biopsies were not executed at the same time. This blood/histologic parallelism concerning Tfh cells was found also in P28 (APDS2). A further fascinating similarity between histologic and blood analysis was seen in P1, who showed serum IgM levels over the upper limit of normal, corresponding to an IgM count in interfollicular zones and GC far above the third quartile of the values registered in 17/20 patients with reassessed nonneoplastic/reactive LPD (in-depth multiparametric histological analysis of P17, P18, P19, and P31 was not performed). An analogous correspondence regarding blood, interfollicular, and GC IgM expansion was found in P28 (APDS1), P31 (APDS2), and P26 (CVID-like); in P27 (APDS2), elevated blood and interfollicular IgM levels contrasted with an almost complete absence of IgM within the GC, while in P28, we detected an increased number of interfollicular and GC IgM+ plasma cells in the face of lower to absent IgG+ plasma cells. Moreover, P1 and P28 both showed an increased number of Tia1+ activated cytotoxic T cells in comparison with CD8^+^ T cells, likely due to persistent EBV replication with viremia >1,000 copies/ml (Table 2).

In P16 and P25 (CVID/CVID-like), we found PTGC-like features in the context of mixed T/B cells and follicular hyperplasia of B cells, respectively.

**Figure S4.**
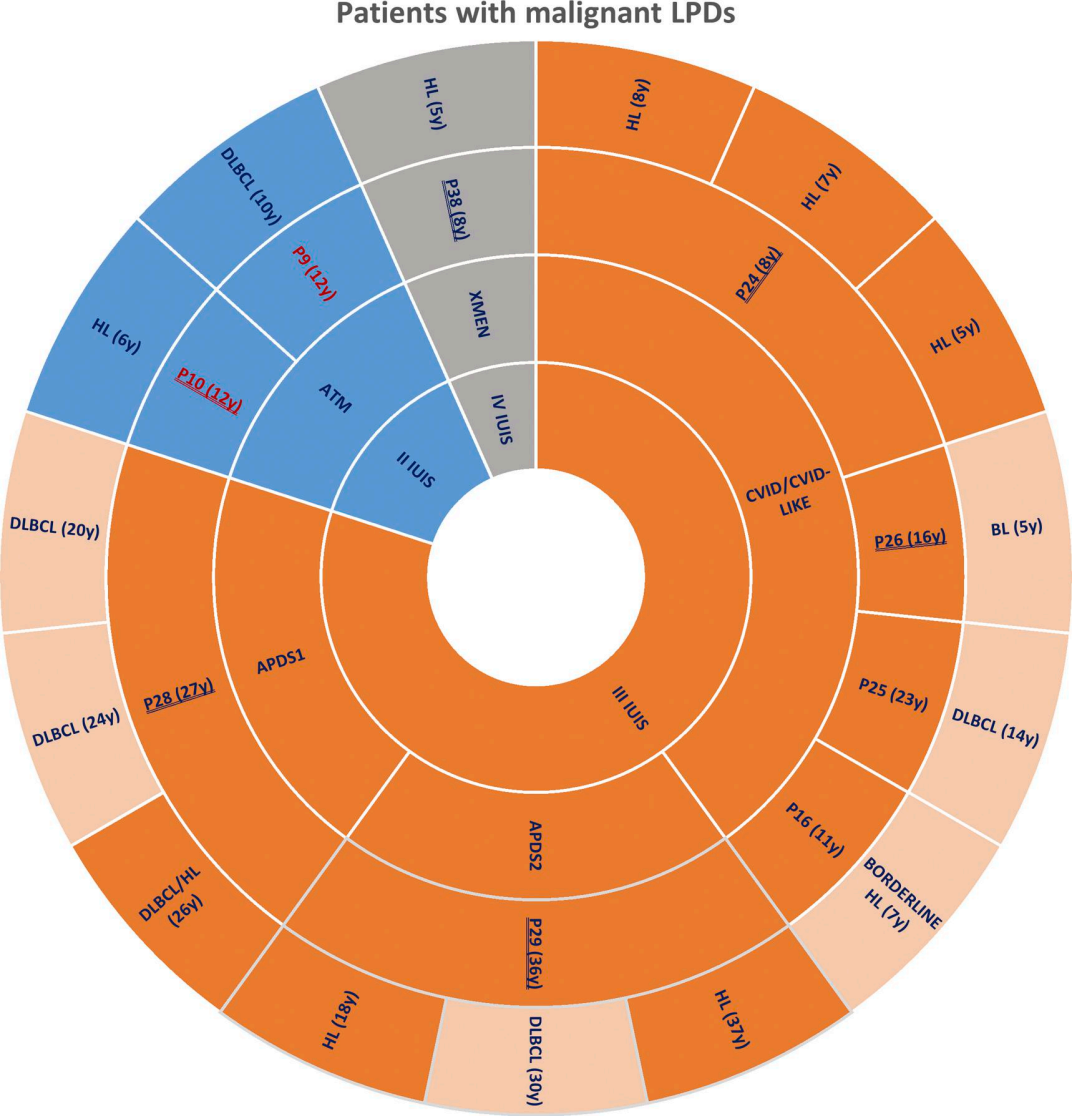
**Patients with overt lymphoid neoplasm (lymphoma).** The innermost ring categorizes patients based on the IUIS classification of IEI. The second inner ring details specific IEI diagnoses. The third inner ring shows the identification number of each patient, reporting in parentheses the age at last follow-up; double-underlined patients are those experiencing EBV chronic replication with a viremia >1,000 copies/ml during follow-up, while red bold patients are those who succumbed to lymphoma. The outermost ring specifies the type of lymphoma, reporting in parentheses the age at overt lymphoid neoplasm (lymphoma) diagnosis. EBER-negative lymphomas are displayed in a lighter shade, distinguishing them from EBER-positive lymphomas, which retain the same color shade as their corresponding internal classification rings.

Among the 38 patients enrolled due to suspected or confirmed IEI-related LPD, nine (24%) were diagnosed with overt lymphoid neoplasms (lymphomas) as depicted in the sunburst diagram (Fig. S4).

Overall, 15 overt lymphoid neoplasms (lymphomas) from nine patients were confirmed upon reanalysis, with EBV-encoded small RNAs (EBER)–positive cHL being the most common (8/15, 53%), followed by diffuse large B cell lymphoma (DLBCL) (5/15, 33%, EBER-positive in a single case), EBER-negative NLPHL (1/15), and EBER-negative Burkitt lymphoma (1/15). CVID was the most frequently associated IEI in terms of both the number of patients affected (4/9, 44%) and total overt lymphoid neoplasm (lymphoma) diagnoses (6/15, 40%)—on par with APDS (6/15, 40%). Lymphoma resulted EBER-positive in 9/15 cases (60%), specifically in the two ATM patients (P9 and P10), in the two APDS patients (P28 and P29), in the XMEN patient (P38), and in a CVID patient whose genetic confirmation for APDS2 is currently ongoing (P24), being associated with persistent EBV infection with a viremia >1,000 copies/ml in 5/6 patients, except for 1 ATM patient (P9). This is in line with the known significant role of EBV in lymphomagenesis, with some cases potentially arising through EBV-independent mechanisms.

Of the nine EBER-positive overt lymphoid neoplasm (lymphoma) cases, eight (89%) exhibited persistent high-load EBV viremia (>1,000 copies/ml) (Fisher’s exact test, P = 0.152; Phi correlation coefficient Φ = 0.395, P = 0.093). In contrast, among 15 nonneoplastic/reactive LPD (excluding P14, P15, P18, P19, and P31, in whom EBER was not assessed), EBER was positive in only two cases (13%) and was associated with high-load viremia in a single case (Fisher’s exact test, P = 1,000; Φ = 0.122, P = 0.649).

However, the classification of the lesions as overt lymphoid neoplasms (lymphomas) versus nonneoplastic/reactive LPD was not significantly associated with persistent high-load EBV viremia (P = 0.153).

For the purposes of the subsequent clinical, immunological, and genetic analyses, patients are grouped according to the centralized histopathologic outcome as nonneoplastic/reactive LPD or overt lymphoid neoplasm (lymphoma). This histology-based grouping is used consistently in all comparative analyses below (immunophenotype, principal component analysis [PCA], genetic yield, therapies, and outcomes).

### Age and signs/symptoms at onset, diagnosis, and follow-up

**Figure 3.**
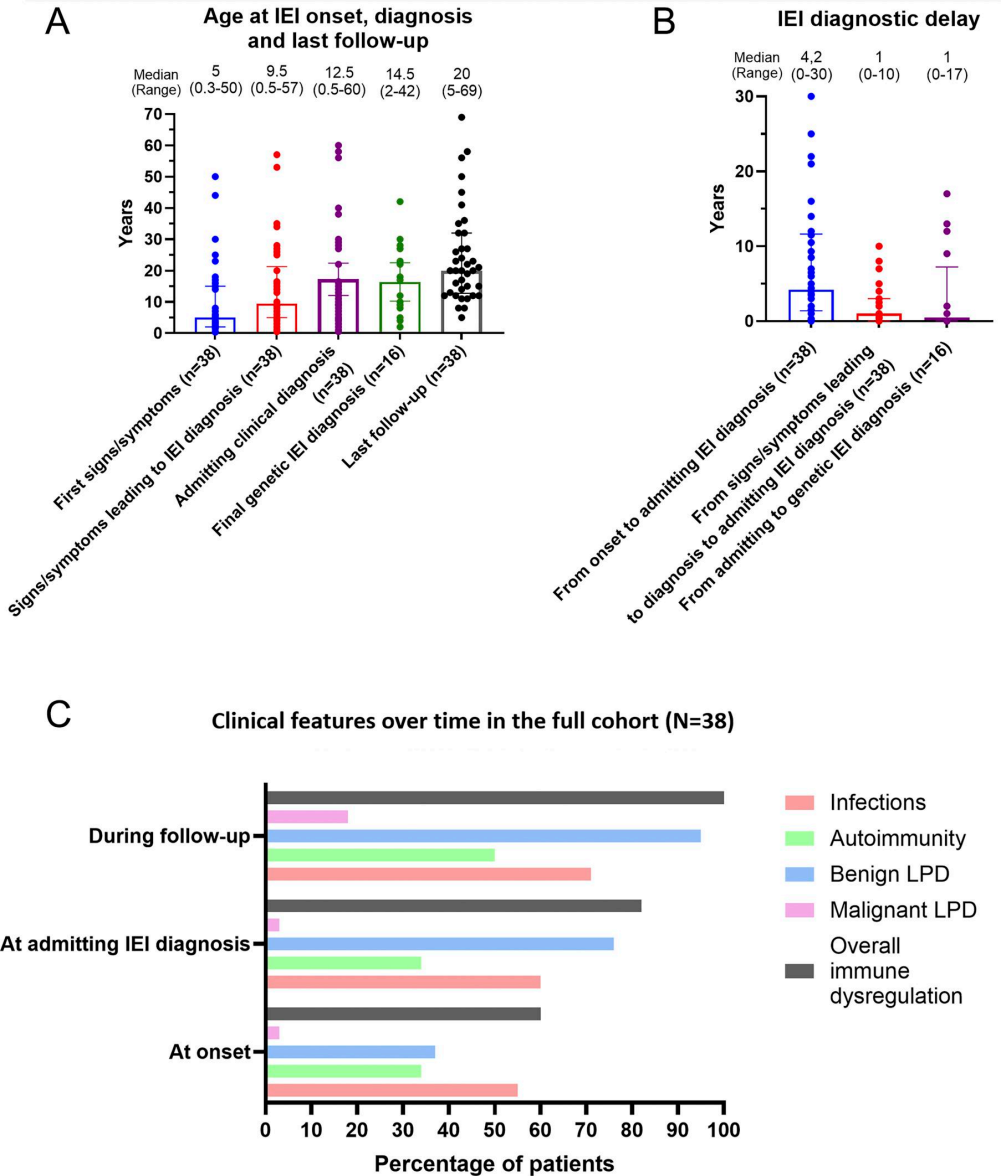
**Illustrates the clinical timeline of the cohort, highlighting significant diagnostic delays and the prevalence of lymphoproliferation and infections from disease onset through follow-up. (** **A and B** **)** Age at IEI onset, diagnosis, and follow-up.** (C) **Clinical features over time in the full cohort (*N* = 38).

The clinical–immunological diagnosis of IEI was made at a median age of 12.5 years (range 2–42)—with a diagnostic delay of 4.2 years (range 0–30) from onset of symptoms, and 1 year (range 0–10) from symptoms that prompted immunological evaluation. A genetic diagnosis was confirmed in 15 patients at the median age of 14.5 years (range 2–42) after a median time of 1 year (range 0–17) from the IEI clinical–immunological diagnosis (Fig. 3, A and B). At the last follow-up, 15 out of 38 patients (39%) were under 18 years of age.

At disease onset, 23 of 38 patients (60%) had immune dysregulation manifestations, predominantly lymphoproliferation (15 of 38 patients, 39%), mostly with nonneoplastic/reactive features (Fig. 3 C).

Lymphoproliferation was the predominant single sign leading to the diagnosis of IEI and occurring during follow-up (Fig. 3 C).

Splenomegaly was diagnosed in 27/38 patients (71%), gastrointestinal or hepatological disorders in 18/38 (47%), inflammatory bowel disease in 5/38 (13%), and liver involvement in 11/38 (29%) (Table S1).

Pulmonary manifestations were also common in the cohort, occurring in 18/38 patients (47%); of note, 8/38 (21%) developed granulomatous lymphocytic interstitial lung disease (Table S1).

21/38 patients (55%) developed infections as onset signs of IEI, of which 15/21 (71%) involved the upper or lower respiratory tract, while 30/38 (79%) experienced infectious episodes at IEI diagnosis or during follow-up, of which 21/30 (70%) were respiratory infections (Fig. 3 C and Table S1).

EBV chronic replication with a viremia >1,000 copies/ml on peripheral blood was detected in 16/38 patients (42%) during follow-up (Table S1).

Overall, overt lymphoid neoplasm (lymphoma) affected 9 of 38 patients (24%) and was preceded by nonneoplastic/reactive LPD in all but one case (P26), whose prior clinical history was incomplete because she was referred to our center only after lymphoma diagnosis and treatment. IEI was clinically diagnosed after lymphoma in 4/9 (44%) cases (P24, P25, P26, and P38) (Fig. 3 C and Table S1).

Interestingly, no patient with ALPS developed lymphoma, while 40% of APDS patients had overt lymphoid neoplasm (lymphoma) (Table S1).

No statistically significant differences were observed between groups with and without overt lymphoid neoplasm (lymphoma) in regard to sex, age at clinical and genetic IEI diagnosis, diagnostic delay from clinical onset, diagnostic delay from onset of alarm signs leading to IEI diagnosis, or time from clinical to genetic diagnosis. Moreover, no statistically significant differences between these two groups were observed in IEI type and signs (infection, autoimmunity, LPD, and specific immune dysregulation manifestations) at either clinical onset, onset of alarm signs leading to IEI diagnosis, during follow-up, or at last follow-up (Table S2).

It is noteworthy that the age at clinical onset was statistically significantly lower in the group with overt lymphoid neoplasm (lymphoma) compared with nonneoplastic/reactive LPD (4 versus 12 years of age; P = 0.012), although this finding did not remain significant after multiple-comparison correction (Bonferroni-adjusted P = 1.788; Benjamini–Hochberg [BH] q = 0.596) (Table S2).

All patients had immune dysregulation during follow-up (Fig. 3 C).

Overall, 4 out of 38 patients (10%) experienced a poor outcome. Specifically, two patients with ATM (P9 and P10) succumbed at the age of 12 to cardiorespiratory arrest secondary to multi-organ failure—caused by norovirus infection in the first case and sepsis in the second. Additionally, two patients with non-genetically defined CID(-like) disorders (P4 and P6) died at the age of 12 following allogenic HSCT and at the age of 56 from *Pseudomonas aeruginosa* sepsis, respectively.

A detailed description of further clinical manifestations can be found in Table S1.

### Immunological workup

Immunological features for each patient are summarized in Table S3. No statistically significant differences in age at blood immunophenotype analysis were found between IEI-related LPD patients with and without lymphoma (P = 0.183).

Comparing the groups with and without overt lymphoid neoplasm (lymphoma), laboratory analysis showed a trend toward lower CD3^+^ (PAN-T) cell percentages (P = 0.072) and lower CD4^+^ helper T cell percentages (P = 0.068), both of which were more frequently below the age-adjusted lower limit of normal (LLN) in the overt lymphoid neoplasm (lymphoma) group, though these differences did not reach statistical significance (P = 0.094 for CD3^+^ T cells %; P = 0.084 for CD4^+^ helper T cells %). A similar nonsignificant trend toward lower CD4^+^ absolute counts was observed in patients with overt lymphoid neoplasm (lymphoma) (P = 0.116). CD4^+^ Tfh cells resulted significantly higher in nonneoplastic/reactive LPD group (P = 0.036); however, this association lost significance after Bonferroni and BH correction (Bonferroni-adjusted P = 5.364; BH q = 1.000). Furthermore, CD8^+^ late effector cytotoxic T cell frequency showed a trend toward higher values in the overt lymphoid neoplasm (lymphoma) group (P = 0.144), accompanied by a trend toward lower CD8^+^ effector memory cytotoxic T cell frequency (P = 0.162). As for CD16^+^CD56^+^ natural killer (NK) absolute count, its reduction under the LLN was significantly more prevalent in the nonneoplastic/reactive LPD group (P = 0.002), although this finding did not remain significant after multiple-comparison correction (Bonferroni-adjusted P = 0.298; BH q = 0.149) (Tables S4 and S5).

As regards serum Ig concentration, IgA showed a trend toward lower values in patients with overt lymphoid neoplasm (lymphoma) compared to those without lymphoma (P = 0.092), while IgM diminishment under the LLN was significantly more prevalent in the nonneoplastic/reactive LPD group (P = 0.002); however, this association lost significance after multiple-comparison correction (Bonferroni-adjusted P = 0.298; BH q = 0.149) (Tables S4 and S5).

### PCA of lymphocyte subpopulations

PCA was performed on the relative frequencies of lymphocyte subsets to explore potential immunophenotypic patterns distinguishing histological findings in terms of malignant evolution from nonneoplastic/reactive LPD to overt lymphoid neoplasm (lymphoma).

**Figure 4.**
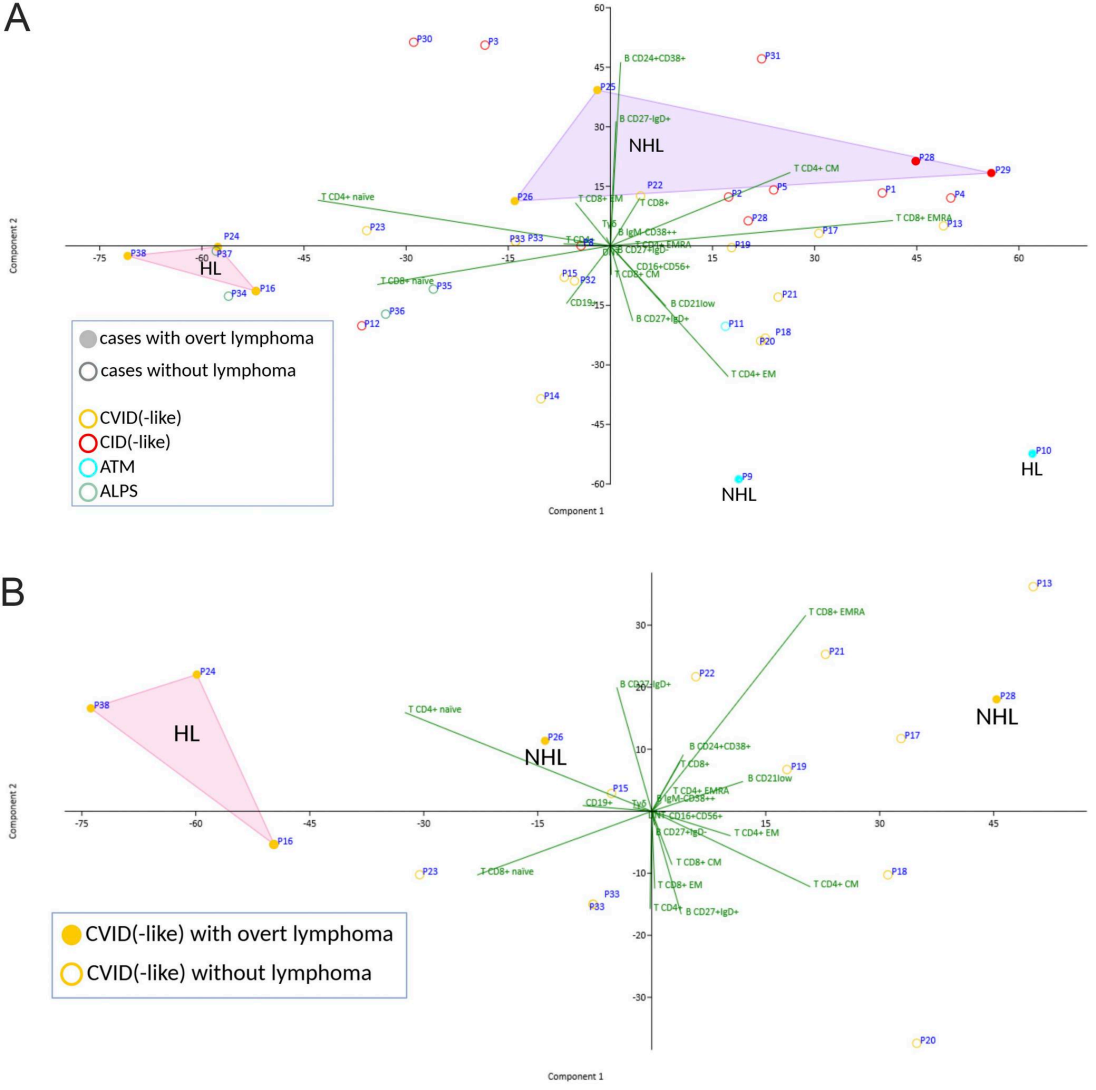
**Displays Principal Component Analysis (PCA) plots of lymphocyte subsets, revealing distinct immunophenotypic clustering by IEI subtype and divergent T-cell patterns between patients progressing to Hodgkin versus non-Hodgkin lymphoma. (A and B)** PCA scatter plots of T and B cell subset frequencies. EMRA, terminl effector memory CD45RA+; EM, effector memory; CM, central memory.

The PCA revealed quite good clustering according to underlying IEI subtype with a clear distinction between patients admitted with CID(-like), ALPS/ALPS-like, and ATM disorders, shown in red, green, and blue dots, respectively (Fig. 4, A and B).

CVID/CVID-like patients, represented with yellow dots, displayed scattered distributions, highlighting known immunologic heterogeneity in this group, as previously described (25, 26). Interestingly, all initially diagnosed CVID patients developing HL, either cHL (P24 and P38) or NLPHL (P16), localized peripherally within the CVID cluster and showed an expansion of naive CD4^+^ and CD8^+^ T cells with reduced central memory CD4^+^ T cells. The same distinctive cluster was confirmed when the PCA was limited to CVID/CVID-like patients (Fig. 4, A and B).

When considered separately for progression to HL and non-HL (NHL), it is noteworthy that the immunophenotypic profiles of patients with LPD that undergo malignant transformation into HL or NHL cluster into almost mirror-image quadrants on the PCA plot. Progression to NHL is associated with an incremental trend in CD4^+^ central memory and CD8^+^ terminal effector memory T cells, whereas progression to HL is characterized by a mirrored defect in the terminal differentiation of naive T cell subsets. This mirror-image separation becomes even more pronounced when the NHL group is restricted to patients who developed DLBCL, after excluding patient P26; P26 is the only case in which it remains unclear whether the overt lymphoid neoplasm (lymphoma) was preceded by a nonneoplastic/reactive LPD, because the prior clinical history was incomplete—she was referred to our center only after lymphoma diagnosis and treatment. Of note, patients P9 and P10 were excluded from the HL versus NHL clustering analysis because they are affected by ATM, in which the pathogenic mechanism leading to neoplasia (defective DNA repair via homologous recombination and chromosomal instability) is well defined and likely distinct from that operating in the other cohort members. Finally, it is noteworthy that, when the cohort of patients with malignant progression of LPD is considered as a whole (both HL and NHL), these cases tend to localize at the extremes of the PCA plot (Fig. 4, A and B).

### Comparison between genetically and non-genetically determined IEI groups

Genetic analysis for each patient is summarized in Table 1.

Among the 38 patients in the cohort, 15 had genetically confirmed IEI (39%), while 23 had no identified genetic etiology (61%). In the group of patients with genetically determined IEI, 10/15 (67%) underwent next-generation sequencing (NGS) and 5/10 (33%) underwent whole-exome sequencing (WES). In the group with genetically indeterminate IEI, 13/23 (57%) underwent NGS and 10/23 (43%) underwent WES. Comparing the groups with overt lymphoid neoplasm (lymphoma) and nonneoplastic/reactive LPD, the rate of genetically determined IEI was higher—although not statistically significant—in the overt lymphoid neoplasm (lymphoma) group (5/9, 56% versus 10/29, 34%; P = 0.4365).

**Figure 5.**
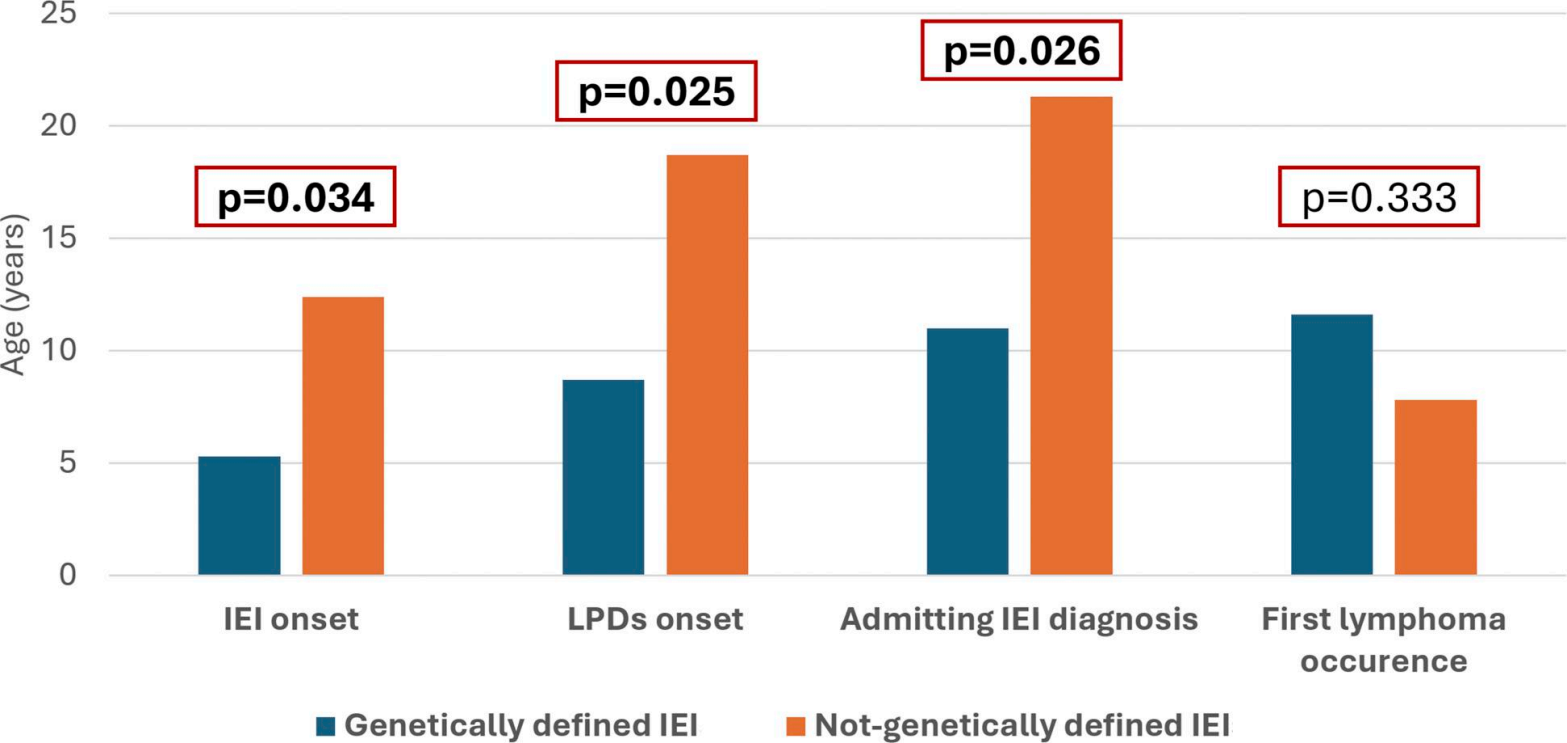
Genetically vs. non-genetically determined IEI groups.

Comparative analysis revealed that patients with a genetically determined IEI had a significantly earlier age at both IEI onset (5 versus 12 years; P = 0.034) and at IEI diagnosis (11 versus 21 years; P = 0.026) compared to those without a genetic diagnosis. Similarly, the age at LPD onset was significantly lower in the genetically determined group (9 versus 19 years; P = 0.025). Interestingly, patients with a genetically defined IEI showed later occurrence of first lymphoma compared to their genetically undefined counterparts, although this difference was not statistically significant (12 versus 8 years; P = 0.333) (Fig. 5).

### Therapies

In terms of supportive care, 27 of the 38 patients (71%) received Ig replacement therapy, reflecting the high prevalence of hypogammaglobulinemia and/or impaired antibody production in this cohort (Table S1).

Furthermore, nine patients (24%) were on prophylactic antibiotic therapy, highlighting the use of infection-prevention strategies, particularly in those with recurrent or severe respiratory infections and T cell defects.

**Figure 6.**
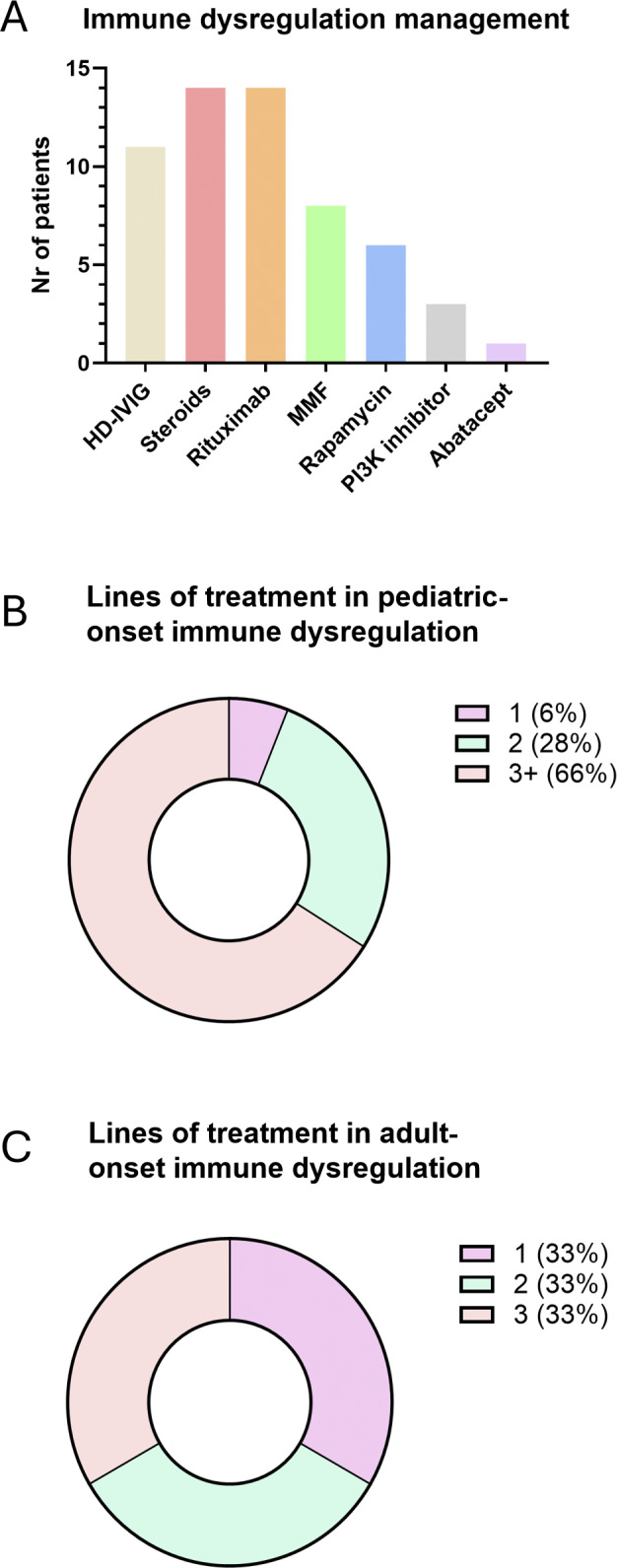
**Details the immunomodulatory therapies administered, demonstrating that patients with pediatric-onset immune dysregulation required more lines of treatment compared to those with adult-onset immune dysregulation. (A–C)** Immunomodulant therapies and lines of treatment. MMF, mofetil mycophenolate; Nr, number.

Immunomodulatory treatments were used in 24 patients (63%), primarily for the management of immune cytopenia or lymphoproliferative manifestations. Notably, 18 of these (75%) required at least one second-line agent due to suboptimal control with initial therapy (Fig. 6, A–C). Immunomodulant regimens included high-dose intravenous Ig (HD-IvIg), steroids, mofetil mycophenolate, rapamycin, rituximab, PI3K inhibitors, and abatacept; HD-IvIg and/or steroids was used as first-line treatments in 17/24 cases (71%) (Table S1). When comparing patients with pediatric- versus adult-onset immune dysregulation, the need for at least three lines of treatment was more frequently observed in the pediatric group (66% versus 33%). A single line of treatment was sufficient in only 6% of pediatric-onset cases, compared to 33% in the adult-onset group (Fig. 6, A–C).

Detailed information regarding the therapies administered for overt lymphoid neoplasm (lymphoma) and the corresponding treatment responses is reported in Table 3.

**Table 3.** Detailed features of the overt lymphoid neoplasm (lymphoma) manifestations in the cohort

​	P9	P10	P16	P24	P25	P26	P28	P29	P38
Sex	F	M	F	M	M	F	M	F	M
IEI type	ATM	ATM	CVID	CVID	CVID-like	CVID-like	APDS1	APDS2	XMEN
Age at IEI onset	2 years	3 years	7 years	3 years	5 years	5 years	6 mo	8 mo	3 years
Age at IEI diagnosis	2 years	3 years	7 years	6 years	16 years	9 years	18 years	30 years	5 years
Type of overt lymphoid neoplasm (lymphoma) (age at diagnosis)	DLBCL (10 years)	cHL (6 years)	Borderline NLPHL (7 years)	• cHL (5 years) • cHL (7 years) • cHL (8 years)	DLBCL (14 years)	BL (5 years)	• DLBCL (20 years) • DLBCL (24 years) • DLBCL/cHL (26 years)	• cHL (18 years) • DLBCL (30 years) • cHL (37 years)	cHL (5 years)
Localization of overt lymphoid neoplasm (lymphoma)	Axillary lymph nodes	Laterocervical and abdominal lymph nodes	Laterocervical lymph nodes	• Laterocervical lymph nodes • Laterocervical, retropharyngeal lymph nodes, palatine tonsils • Laterocervical, retropharyngeal lymph nodes, palatine tonsils	Axillary, pre and paravertebral abdominal lymph nodes; lumbar vertebral bone	Laterocervical lymph nodes and bone (tibia, femur, lumbar vertebra, skull)	• Duodenum • Cecum • Cecum	• Laterocervical lymph nodes • Colon • Laterocervical lymph nodes	Laterocervical lymph nodes
EBER association with overt lymphoid neoplasm (lymphoma)	Yes	Yes	No	• Yes (cHL) • Yes (cHL) • Yes (cHL)	No	No	• No (DLBCL) • No (DLBCL) • Yes (DLBCL/HL)	• Yes (cHL) • No (DLBCL) • Yes (cHL)	Yes
Treatment	6 R-CHOP cycles	• Steroid and rituximab, then 5 VAMP cycles (cHL)	3 CVP cycles	• 2 OEPA + 1 COPDAC cycles (cHL) • 8 brentuximab cycles (cHL) • 4 BeGEV cycles → 4 brentuximab + bendamustine cycles	AIEOP LNH97 protocol, R4 group +2 R-ICE	AIEOP LNH97 protocol	• 3 R-CHOP + 2 CHOP cycles (DLBCL) • DLBCL • Rituximab + ibrutinib + bendamustine (DLBCL/cHL)	• 3 ABVD cycles + radiotherapy (cHL) • 5 R-CHOP cycles + 2 rituximab infusions (DLBCL) • cHL (16 brentuximab cycles)	2 OEPA + 4 COPDAC + 11 rituximab cycles
Outcome	Remission	Remission	Remission	Remission	Remission	Remission	Remission	Stable	Remission
EBV chronic viremia	No	Yes	No	Yes	No	No	Yes	Yes	Yes
Status	Deceased	Deceased	Alive	Alive	Alive	Alive	Alive	Alive	Alive
Age of death	12 years	12 years	/	/	/	/	/	/	/
Cause of death	MOF triggered by sepsis	MOF due to norovirus infection	/	/	/	/	/	/	/

ABVD, adriamycin, bleomycin, vinblastine, dacarbazine; AIEOP LNH97, Italian Association of Pediatric Hematology and Oncology Non-Hodgkin Lymphoma 1997 protocol; BeGEV, bendamustine, gemcitabine, vinorelbine; BL, Burkitt lymphoma; CHOP, cyclophosphamide, doxorubicin, vincristine, prednisolone; COPDAC, cyclophosphamide, vincristine, prednisolone, dacarbazine; CVP, cyclophosphamide, vincristine, prednisone; F, female; M, male; MOF, multi-organ failure; MZL, marginal zone lymphoma; OEPA, vincristine, etoposide, prednisone, doxorubicin; R-CHOP, rituximab, cyclophosphamide, doxorubicin, vincristine, prednisolone; R-ICE, rituximab + ifosfamide, carboplatin, etoposide; VAMP, vinblastine, adriamycin, methotrexate, prednisone.

## Discussion

### The clinical, immunohistopathological, and genetic landscape of LPD in IEI

LPD have increasingly been recognized as a frequent and relevant manifestation in patients with IEI, either as a presenting feature or as a later complication of immune dysregulation.

In this study, 39% of IEI patients with LPD had a genetically confirmed IEI, while the remainder were genetically indeterminate. Interestingly, significantly earlier clinical onset—particularly with respect to LPD—occurred in genetically determined, monogenic IEI, reinforcing the need for prompt immunologic referral, close longitudinal monitoring, and possibly early genetic evaluation in pediatric patients presenting with recurrent infections or immune dysregulation—especially in terms of LPD, as reported in larger cohort of IEI patients (7, 27, 28).

Moreover, pediatric-onset IEI correlated with a more refractory clinical course: two-thirds of pediatric patients treated for immune dysregulation required at least three lines of treatment compared to one-third of adult-onset cases. This suggests an intrinsically aggressive phenotype in early-onset IEI, reinforcing the clinical value of timely diagnosis and individualized management strategies (29, 30).

Despite early clinical manifestations, a substantial diagnostic delay was observed for IEI. The mixed-care setting—pediatric and partially adult—across the two recruiting centers may have made this delay more pronounced since in the adult context awareness of IEI is usually lower than in pediatric care (Table S2).

Over half of the genetically indeterminate IEI patients (57%) underwent non-informative NGS panels, suggesting a role for periodic additional and deeper genetic evaluations in genetically unsolved IEI over time. Furthermore, the lower genetic diagnostic rate in the nonneoplastic/reactive LPD group could be due to the dominant prevalence of CVID(-like) disorders (45%), in which genetic IEI diagnoses are less frequently established (31).

In the genetically confirmed, monogenic IEI group, lymphoma occurred at a later age compared to the genetically undefined IEI group (12 versus 8 years), largely reflecting the presence of APDS patients, which accounted for 40% of the genetically determined IEI group with lymphoma, and are known for later-onset malignancy (19).

The current uncertainty regarding the intrinsic malignant potential of LPD and the factors influencing this risk—combined with the histopathological overlap between nonneoplastic/reactive LPD and overt lymphoid neoplasms (lymphomas) (6, 32, 33)—further underscores the need for comprehensive research to guide clinical decision-making and improve patient outcomes (34). In our cohort, 35% of patients who underwent excisional lymph node biopsy required multiple procedures over time, with a mean of approximately three biopsies per patient. This finding highlights the often prolonged and invasive diagnostic journey due to the lack of standardized clinical pathways.

Table 4 summarizes a pragmatic, stepwise diagnostic algorithm and checklist for the assessment of LPD in patients with suspected IEI, integrating clinical red flags, baseline laboratory/virology/imaging workup, preferred tissue-sampling and minimum histopathology tests, a staged genetic-testing pathway, and multidisciplinary decision points—recommendations derived from our cohort experience and current consensus practice.

**Table 4.** Diagnostic algorithm for IEI-related lymphoid proliferations

Step	Action/Tests	Key decision points
1. Red flags	• Persistent lymphadenopathy, hepatosplenomegaly, or organ lymphocytic infiltration >3 mo ± • Recurrent/unusual infections, early-onset disease, syndromic features, family history of IEI/malignancy • Chronic or high-load EBV viremia (quantitative EBV PCR)	If any red flag → initiate full pathway: Baseline investigations and consider excisional biopsy when feasible
2. Baseline investigations	Clinical: Detailed history (infections, autoimmunity, family), full exam (nodes, spleen, syndromic stigmata) Laboratory (minimum panel): CBC ± reticulocytes; LFTs; creatinine; LDH; quantitative IgG (±subclasses), IgA, IgM; serum/urinary protein electrophoresis	Purpose: Identify organ dysfunction, immune phenotype, and markers of active disease to guide biopsy timing and therapy
2. Immunology	Extended lymphocyte subsets (T/B/NK, including circulating Tfh, naive/memory markers)	Abnormal profiles (e.g., APDS signatures, hypogammaglobulinemia) raise suspicion for specific IEI and inform genetic panel choice
2. Virology	Quantitative EBV PCR (blood) ± other viral PCRs (CMV, HHV-6)	Persistent/high EBV load supports EBV-driven LPD and prompts urgent tissue workup and multidisciplinary review
2. Imaging	Ultrasound/CT for lymph nodal assessment; consider PET-CT to select biopsy site and for staging/response assessment	PET helps choose most metabolically active node for excision when multiple sites exist
3. Tissue strategy	Primary: Excisional lymph node biopsy (preserve architecture). If excision impossible: Obtain a large core biopsy (avoid small cores if diagnosis uncertain)	Excisional biopsy maximizes diagnostic yield and accuracy of histologic subclassification
3. Standard histopathology panel	H&E (full sections); broad IHC (e.g., CD20, CD3, CD4, CD8, CD30, PAX5, MUM1/IRF4); EBER in situ hybridization (mandatory if EBV suspected); clonality assays (IGH/IGK and TCR rearrangement PCR); additional stains (ALK, CD10, BCL2/BCL6) guided by morphology	Combine morphology, EBER, and clonality to distinguish nonneoplastic/reactive LPD from lymphoma; retain tissue for ancillary tests
3. If histology indeterminate	Convene rapid multidisciplinary review (pathology, immunology, hematology/oncology, genetics, ID); correlate EBV load, peripheral immunophenotype, and genetics; consider close imaging/clinical follow-up or repeat excisional biopsy	Integrated review may change classification and avoid misdiagnosis; repeat excision if clinical progression
3. Genetic testing	Stage 1: Targeted NGS IEI panel (genes related to lymphoproliferation—e.g., APDS/PIK3CD, CTLA4, LRBA, RAG1/2, PRF1, etc.); send concurrently with biopsy Stage 2: If Stage 1 negative and suspicion persists → WES ± CNV analysis; consider trio testing and targeted search for somatic/mosaic variants Stage 3: If unresolved → WGS, structural variant analysis, repeat bioinformatic reanalysis periodically	Early genetics informs treatment (e.g., targeted agents, chemo modification, HSCT consideration). Centralize genetic reports and reanalyze as knowledge evolves
4. Multidisciplinary integration	Convene MDT (I+immunology, hematopathology, hemato-oncology, genetics, infectious disease) to integrate: Clinical course, EBV kinetics, histology, clonality, and genetics	If overt lymphoid neoplasm (lymphoma) confirmed: Oncology-led therapy with IEI-informed adaptations (infection prophylaxis, chemo-dose considerations), early HSCT discussion when indicated. If nonneoplastic/reactive but high-risk features: Consider targeted/bridge therapy (e.g., PI3Kδ inhibitor for APDS), close surveillance, and formal HSCT evaluation for refractory cases
4. HSCT considerations	HSCT may be curative for many severe IEIs and some IEI-associated LPDs. Consider donor options including matched, mismatched, and HLA-haploidentical platforms (TCRαβ/CD19 depletion or post-transplant cyclophosphamide) where applicable	Balance curative potential against risks (graft failure, GVHD, infections, TRM). Use targeted agents (e.g., leniolisib) as bridging therapy when appropriate; long-term safety/oncologic impact requires continued surveillance
Minimum follow-up schedule	Clinical review and EBV PCR: Baseline, then every 4–12 wk during active disease/therapy; extend interval in stable disease Imaging: PET-CT for staging/response; ultrasound for surveillance Repeat excisional biopsy for progression or new worrisome features	Tailor frequency to disease activity, treatment, and EBV kinetics

Diagnostic algorithm for lymphoproliferative disorders. The pathway illustrates a stepwise approach: identifying clinical red flags (step 1) triggers investigations that progress from baseline assessments (step 2) to ever more specialized and integrated testing and management (steps 3 and 4). ALK, anaplastic lymphoma kinase; BCL, B cell lymphoma; CBC, complete blood count; CMV, cytomegalovirus; CNV, copy number variation; CT, computed tomography; GVHD, graft-versus-host disease; H&E, hematoxylin and eosin; HHV-6, human herpesvirus 6; ID, infectious disease; IGH/IGK, immunoglobulin heavy chain/immunoglobulin kappa light chain; IHC, immunohistochemistry; LDH, lactate dehydrogenase; LFTs, liver function tests; MDT, multidisciplinary team; PCR, polymerase chain reaction; PET-CT, positron emission tomography-CT; PI3Kδ, phosphoinositide 3-kinase delta; TCR, T cell receptor; TRM, transplant-related mortality; WGS, whole-genome sequencing.

### Pathophysiological mechanisms of overt lymphoid neoplasm (lymphoma) evolution and the role of EBV

The overt lymphoid neoplasm (lymphoma) evolution of LPD in IEI patients arises from a complex interplay of intrinsic genetic defects and extrinsic factors such as persistent EBV-driven immune dysregulation (32, 35).

Indeed, patients with ATM (P9 and P10) developed early-onset EBV-related overt lymphoid neoplasm (lymphoma) through a route dominated by intrinsic genomic instability, acting as the primary oncogenic driver and EBV infection serving as an additional proliferative trigger (36). A similar lymphomagenic trajectory is suggested by XMEN syndrome—caused by *MAGT1* mutations—disrupting TCR signaling and NKG2D expression on NK and CD8^+^ T cells, sustaining chronic EBV viremia and early lymphoma, as exemplified by P38 and his paternal uncle, carrying the same genetic defect and developing multiple EBV-related lymphomas at a young age (37).

Conversely, APDS mutations in *PIK3CD*/*PIK3R1* hyperactivate PI3Kδ, impairing CD8^+^ T cell function and class switching in B cells. This undermines EBV control and thus fosters B cell malignancy over time, with a greater impact of extrinsic factors on overt lymphoid neoplasm (lymphoma) evolution (38)—as reported in two APDS patients (P28 and P29) with later-onset lymphomas and in other APDS cohorts (19, 39, 40, 41, 42, 43, 44, 45, 46).

Although differences in EBER expression and high-load viremia between the overt lymphoid neoplasm (lymphoma) and nonneoplastic/reactive LPD groups did not reach statistical significance, the trends highlight the role of EBV in overt lymphoid neoplasm (lymphoma) transformation.

These findings highlight how diverse IEI genotypes intersect with chronic EBV to accelerate lymphomagenesis through shared and distinct routes (47). Importantly, although some lymphomas arising in immunocompromised hosts may develop with fewer oncogenic hits and in some cases could be more chemosensitive, therapeutic decisions in patients with IEI must be individualized (48, 49). Patients with DNA-repair disorders (e.g., ATM or Nijmegen breakage syndrome) and V(D)J recombination disorders (e.g., LIG4 and ARTEMIS deficiency) are at substantially increased risk of severe treatment-related toxicity from DNA-damaging agents and ionizing radiation and therefore frequently require adaptation of standard regimens (50, 51). At the same time, reductions in chemotherapy relative dose intensity have been associated with inferior oncologic outcomes in multiple tumor settings, especially hematological malignancies (52, 53, 54, 55), although some case series, including IEI patients, report favorable outcome for low-intensity therapy in overt lymphoid neoplasms (lymphomas) (56, 57); several expert consensus documents and case series therefore emphasize the need to balance toxicity avoidance with the risk of undertreatment and to manage these patients within multidisciplinary teams at experienced centers, considering alternatives such as targeted agents, immunotherapy, or adapted HSCT strategies when appropriate (49, 51). Allogeneic HSCT remains the only established curative option for many severe IEI and is increasingly considered for selected adolescents and adults with progressive immune dysregulation, life-threatening infections or recurrent/multisite overt lymphoid neoplasm (lymphoma) (10). In our series, only one patient (P4) underwent allogeneic HSCT and subsequently died; one additional patient (P27) received autologous HSCT. Although allogeneic HSCT can be curative for APDS in selected patients, contemporary series report both encouraging disease control in many transplanted individuals and substantial transplant-related risks (graft failure, viral reactivation, graft-versus-host disease, and treatment-related mortality) that must be weighed in each case (58). Decision-making therefore depends on disease severity (refractory lymphoproliferation, recurrent or multiple-site overt lymphoid neoplasm [lymphoma], severe immune cytopenias, or organ-threatening infection), patient age and comorbidity, prior therapies and organ function, donor availability, and center expertise (58, 59); where feasible, targeted therapies (for example PI3Kδ inhibitors) or other bridging strategies may be used to control disease prior to HSCT or, in milder phenotypes, to avoid transplant altogether (19). In our cohort, P28 received PI3Kδ-directed therapy with clinical benefit, including a reduction in lymphadenopathy and immune dysregulation, and partial restoration of T cell subsets distribution. Although his history of recurrent and multisite overt lymphoid neoplasms (lymphomas) had previously prompted consideration of allogeneic HSCT, this option was ultimately deferred due to his late genetic diagnosis in early adulthood, existing comorbidities, lack of a matched HLA donor, and the sustained clinical response achieved with targeted therapy. Although long-term follow-up of PI3Kδ inhibitors in APDS is still limited, and theoretical concerns (e.g., drug-related effects on AID expression) on its intrinsic lymphomagenesis risk, albeit debatable (60), have been raised (61), leniolisib has demonstrated marked reductions in lymphadenopathy and improvement in immunophenotypic markers in clinical trials and therefore may serve as a valuable bridging option to HSCT to control lymphoproliferation prior to definitive transplantation (62).

Recent work also documents promising outcomes with HLA-haploidentical HSCT platforms for IEI (thereby expanding donor options when matched donors are unavailable), making haploidentical approaches an important consideration in selected patients (63, 64, 65, 66, 67, 68, 69, 70, 71, 72, 73, 74).

Therefore, vigilant surveillance of both well-recognized high-risk and low/undefined-risk IEI is essential to intercept overt lymphoid neoplasm (lymphoma) progression and optimize outcomes.

### Masquerading malignancy: Diagnostic pitfalls of Hodgkin-like lymphoproliferation in IEI

In our cohort, 31% of patients initially diagnosed with hematologic malignancies were reclassified as nonneoplastic/reactive following IEI diagnosis and histologic reassessment. Moreover, P16, suffering from CVID and originally diagnosed with lymphoma, exhibited a borderline case between NLPHL and mixed hyperplasia with a PTGC-like pattern (Fig. 2).

As recently reported in different group of IEI patients—such as IL2RG-deficient SCID and CVID—nonneoplastic/reactive lymphoid hyperplasia may mimic cHL both histologically and immunophenotypically (3, 75, 76, 77). This diagnostic ambiguity has serious therapeutic implications, as illustrated by P27 who was exposed to unnecessarily aggressive treatment.

As expected, the diagnostic reassessment largely involved HL (cHL in 2/5 cases and NLPHL in one additional case), suggesting diagnostic overcalling in some instances. From a pathophysiologic perspective, HL—whether associated with IEI or not—may in certain contexts reflect pronounced immune dysregulation that is difficult to distinguish from overt neoplasia by reason of the generally low tumor cell burden, the prominence of inflammatory tumor-infiltrating cells, and the resultant variability in cellular composition. Crucially, this entity generally exhibits favorable responses to low-intensity chemotherapy and immunomodulatory approaches, with the latter increasingly playing a pivotal role (77, 78, 79).

Interestingly, during the revision process of the manuscript, patient P29 (APDS2) developed additional laterocervical, axillary, and crural lymphadenopathies, which were initially diagnosed as cHL and treated with pembrolizumab. Histopathological re-evaluation revealed a largely preserved lymph node architecture, with B cell follicles positive for CD20 and a paracortical area rich in T cell elements (CD3^+^/PD1^+^), admixed with large blast-like cells variably positive for CD30, CD15, CD20, CD79a, and PAX5 (weak to moderate), and negative for LMP1 and EBV by in situ hybridization. Marked sinus histiocytosis with abundant DPL1^+^ macrophages was also observed. Overall, the lymph node structure was preserved, showing features of pronounced immune activation that did not fulfil the diagnostic criteria for cHL, thus reclassifying the case as an immunodeficiency-associated LPD. Evaluation of pembrolizumab discontinuation and initiation of leniolisib therapy is currently ongoing.

When patients with LPD are stratified by eventual malignant outcome (HL versus NHL), their immunophenotypic profiles cluster into largely distinct regions of PCA space, an observation consistent with recent multimodal, single-cell, and flow-cytometry atlases of nodal B cell NHL showing entity-specific T cell infiltration patterns and clear PCA-based separation (80). Aggressive entities such as DLBCL are characterized by depletion of CD4^+^/CD8^+^ naive T cells and enrichment of PD-1^+^/effector memory/exhausted T cell populations, providing a mechanistic basis for the NHL-associated shift toward central/effector memory phenotypes observed in our data, although we analyzed peripheral blood rather than nodal tissue immunophenotypes (80). Work in chronic lymphoproliferative conditions such as chronic lymphocytic leukemia, pathobiologically much more closely related to NHL rather than HL spectrum, documents progressive skewing of the peripheral and nodal T cell compartment from naive toward differentiated effector/memory phenotypes, which lends additional plausibility to the NHL-associated memory/T effector enrichment we report (81). As for HL, interestingly, in CVID/CVID-like patients developing either cHL (P24 and P38) or NLPHL (P16), PCA revealed a distinct cluster defined by expanded naive CD4^+^ and CD8^+^ T cell pools and diminished central memory CD4^+^ T cells—contrasting with immunocompetent patients with cHL, where chronic activation depletes naive T cells in favor of effector/memory and regulatory subsets (82) (Fig. 4, A and B). This divergence likely reflects CVID-associated immune dysregulation, in which reduced immunologic surveillance and defective memory formation create a permissive milieu for overt lymphoid neoplasm (lymphoma) transformation—although other extrinsic factors, including age, could influence this different immunophenotypic pattern.

Collectively, these data suggest a unique immunopathogenic pathway for cHL/NLPHL in CVID(-like) disorders—driven by altered T cell differentiation—and indicate that such immunophenotypic subclusters could serve as early indicators of malignancy risk in genetically undefined IEI patients.

### Immunophenotypic and histological correlates of malignancy and underlying IEI

In the overt lymphoid neoplasm (lymphoma) LPD cohort, we observed a trend toward lower mean percentages of CD3^+^ (PANT-T), CD4^+^ helper, and CD8^+^ effector memory T cells, accompanied by a trend toward higher frequencies of CD8^+^ late effector memory T cells in peripheral blood mononuclear cells (Tables S3 and S4). These alterations suggest that compromised T cell immunity may be a lymphoma risk factor in IEI-associated LPD, given the role of T cells in EBV control (47, 83). Furthermore, the shift toward late effector memory CD8^+^ T cells points to an immunosenescent-like phenotype as a potential predictor of overt lymphoid neoplasm (lymphoma) transformation, consistent with Bateman et al.’s findings of increased CD8^+^ terminal effector memory in CVID patients with polyclonal LPD and autoimmune cytopenia (26).

Our data reveal concordance between circulating immunological markers and lymph node histopathology in nonneoplastic/reactive LPD, potentially aiding in the diagnosis of IEI. Consistent with findings in APDS (84), certain patients—specifically P1 (CID), P22 (CVID), and P28 (APDS1)—exhibited increased PD-1^+^ Tfh cells within GCs, mirroring Tfh expansion in peripheral blood. This phenomenon is associated with immune dysregulation and suggests a propensity toward autoimmunity or B cell neoplasia (Table 2) (85, 86). Additionally, three APDS patients (P27, P28, and P31) displayed elevated serum IgM levels corresponding to increased IgM deposits in interfollicular areas and GCs. Notably, patient P28 with nonneoplastic/reactive LPD showed a reduced or absent number of IgG+ plasma cells compared to expanded IgM+ plasma cells, along with ill-defined GCs, absence of follicle mantles, and hyperplasia of monocytoid B cells. These features, previously documented in APDS patients (87), underscore histological–immunological correlations that may guide IEI classification and serve as diagnostic clues for suspecting an underlying IEI in undefined LPD.

Although Castleman-like and PTGC-like features were each observed in a minority of cases (12%), these histopathological findings may serve as potential diagnostic clues for pathologists to consider an underlying IEI in the setting of LPD.

ALPS can associate serum hypergammaglobulinemia with nodal IgG4^+^ plasma cell hyperplasia resembling multicentric Castleman disease (88), but P37 exhibited hypergammaglobulinemia and paracortical T cell expansion without GC IgG4 upregulation, likely reflecting both his young age and the non-germinal nature of his *FAS* pathogenic variant.

In lymph node biopsies from patients with LPD, a constellation of histopathological findings can raise suspicion for an underlying IEI. In our series, interfollicular PD-1 expression was universal (16/16), and an interfollicular CD4:CD8 ratio skewed toward CD8^+^ T cells was very common (14/16, 87.5%), whereas increased CD8^+^ T cells within GC—an abnormal finding because GC normally contain very few/none—was seen in 25% (4/16). Aggregates (>3) of CD303/CD123+ plasmacytoid dendritic cells (pDCs) were present in 62.5% (10/16) but may also occur in autoimmune disorders. Among CVID/CVID-like cases, PTCG-like features were observed in 2/8 (25%), and increased interferon regulatory factor 4 (IRF4)+ plasma cells within GC—normally restricted to a few peripheral GC exiting cells—were seen in 3/8 (37.5%). None of these features is pathognomonic for IEI, but their presence, particularly when multiple clues co-occur or when clinical signs of immune dysregulation are present, should prompt multidisciplinary review and early genetic testing (targeted IEI panel with escalation to WES/whole genome sequencing (WGS)/copy number variation (CNV) and mosaicism-directed analyses if negative) to avoid diagnostic delay or misclassification (Table S6).

### Strengths and limitations

This study’s novelty lies in its detailed immunological and histological stratification of IEI-related LPD by malignancy status and in identifying preliminary immunologic and histopathologic associations that could suggest future biomarker research.

We acknowledge limitations related to cohort size and potential referral bias, as the cohort does not capture patients with LPD who were never referred to immunology. Historical variations in multidisciplinary referral practices over the 1996–2022 period may also have contributed to the modest size of the collected cohort. These factors may limit the generalizability of our findings and are important to consider when interpreting the results.

Genetic testing was not uniform across the cohort because patients were enrolled at two different centers and tested over an extended time frame. The heterogeneity of testing platforms and gene content may have impaired the ability to detect pathogenic variants outside the interrogated gene lists. We therefore acknowledge a potential underestimation of the true genetic diagnosis rate and recommend reanalysis or more comprehensive testing (e.g., WES/WGS and CNV analysis) in unsolved cases as knowledge and resources evolve.

Limited tissue availability, incomplete EBER assessment in a few benign cases, and sampling or timing effects (such as chemotherapy or immunomodulatory treatments given before or between biopsies) may have masked the pathologic features indicative of EBV-positive polymorphic LPD (for example, by reducing detectable EBV signal) and led to a nonspecific reactive picture.

Eventually, we acknowledge the subjectivity inherent in manual cell counts used to compare B and T cell populations. Nevertheless, automated, whole-slide quantification of inflammatory cells is not readily applicable to routine practice and, because immunostaining is heterogeneously distributed across tissue sections, is unlikely to provide a clear diagnostic advantage. Moreover, several non-IEI inflammatory or autoimmune disorders may exhibit overlapping morphologic features—e.g., abundant interfollicular plasma cells, aggregates of pDCs, and prominent interfollicular PD-1^+^ T cells. Despite these limitations, we included a composite panel of representative immunostainings corresponding to the heat-map reported in Table 2 (Figs. S1 and S2).

### Conclusion

Effective management of IEI-related and complex LPD demands close collaboration among immunologists, hematologists, and pathologists to integrate clinical presentation, serology, histology, immunohistochemistry, clonality assays, EBV status, and genetic findings. Indeed, reliance on histopathology alone risks misclassification, while atypical cases should prompt re-biopsy and multidisciplinary genetic reassessment to avoid overtreatment.

The field urgently needs standardized diagnostic algorithms and predictive models, supported by prospective studies in larger IEI cohorts with temporally matched serial LPD excisional biopsies and immune profiling—both at LPD onset and over the disease course—to refine risk stratification of overt lymphoid neoplasm (lymphoma) evolution of lymphoproliferation and improve outcomes.

## Materials and methods

### Study design

This retrospective observational study included 85 patients suffering from LPD who were seen at the Immunology Clinic of the IRCCS University Hospital of Bologna and the Unit of Clinical Immunology and Vaccinology of the IRCCS Bambino Gesù Children’s Hospital in Rome, Italy, between January 1996 and December 2022. Subsequently, 38/85 (45%) received a clinical and/or genetic diagnosis of an underlying IEI—either suspected or confirmed.

#### Inclusion criteria for enrollment

• Diagnosis of LPD, either as an initial or subsequent clinical manifestation, defined as persistent lymphadenopathy, hepatosplenomegaly, or lymphocytic organ infiltration for >3 mo, with or without chronic or significant EBV infection, confirmed by standard clinical, laboratory, and radiological procedures ruling out non-EBV infection and malignancy; chronic or significant EBV infection—as clarified by Forbes et al. (7)—is characterized either by recurrent or persistent EBV viremia for >3 mo, invasive EBV disease, or EBV DNA viremia >100,000/μl.
• Diagnosis of IEI according to the criteria of the European Society for Immunodeficiency (89) or the IUIS (1, 2).
• Informed written consent by patient and/or legal guardian.

#### Exclusion criteria

• Patients never referred for immunological evaluation or lacking immunological follow-up.
• A known non-EBV-related infectious cause of LPD.
• An isolated confirmed histological diagnosis of lymphoma, unless the patient presented—before or after the overt lymphoid neoplasm (lymphoma)—with LPD in the context of an underlying clinical and/or genetic diagnosis of IEI—either suspected or confirmed.
• Secondary immunodeficiencies, namely HIV infection or solid organ transplantation.

A comprehensive report was produced based on an in-depth characterization of clinical, laboratory, histopathological, and genetic data. Recruitment was performed during regular medical checkups according to clinical practice. The study was conducted in accordance with the Declaration of Helsinki, and the protocol was approved by the Ethics Committee of the IRCCS University Hospital of Bologna (Project identification ID-TYPE 424/2023/Oss/AOUBO).

### Clinical course

The main items considered to describe the cohort’s clinical features were IEI clinical onset, type of IEI clinical and genetic diagnosis and age at diagnosis, diagnostic delay from clinical onset, infectious susceptibility, chronic or significant EBV infection as defined by Forbes et al. (7), immune dysregulation symptoms (allergy, autoimmunity, hyper-inflammation, LPD, and malignancy), and immunomodulant therapy (for details, see Tables 1 and S1).

### Immunological assays

Laboratory investigations focusing on immunological assets included leukocyte formula (Sysmex XN-20) and serum Ig levels (IgG and subclasses, IgA, IgM, and IgE) through an immunoturbidimetric method (Beckman Coulter).

Extended lymphocyte phenotyping was performed through multiparametric flow cytometry, and samples were acquired with a BD FACSCanto II (Becton-Dickinson). Flow cytometric data analysis was carried out with FlowJo v9.3 software (BD Life Sciences). The immunological subsets are included in Tables 3, S3, and S4.

Immunological data were age referenced and gathered in the absence of evidence of infections at the time of blood sampling, prior to Ig replacement therapy, and >3 mo from vaccine administrations, and >6 mo after the end of immunomodulatory treatments that could potentially affect numerical distribution and differentiation of lymphocyte subsets.

### Histological findings

Tissue from patients suffering from an LPD was not available for re-evaluation in 13/38 patients (34%); in one patient (P8), small paratracheal lymph nodes were not suitable for reassessment, while in 12 cases, no lymph node biopsy/excision was performed because standard clinical, laboratory, and radiological procedures were sufficient to rule out non-EBV-related infectious and overt lymphoid neoplasm (lymphoma). LPD tissue was available for re-evaluation in 25 of 38 patients (66%). Of these, 21 specimens—excluding those from P10, P25, P26, and P38—all obtained by excisional lymph node biopsy to maximize preservation of nodal architecture and improve the reliability of morphologic assessment, were re-examined and reclassified according to the 2024 WHO-HAEM5 (6).

(6) For the cases classified as nonneoplastic/reactive hyperplasia, histological analysis focused on the descriptions of the B cortical and T paracortical compartments. The first evaluation was whether the hyperplasia was due mainly to B cells (follicular hyperplasia), T cells (paracortical hyperplasia), or both (mixed hyperplasia).

For B cell follicles any Castleman-like features were described—defined by GC atrophy, follicular dendritic cell hyperplasia, hyperplastic mantle zone with concentric disposition (so-called “onion skin” appearance), and perpendicular vessels penetrating the GC (“lollipop” appearance) in at least 20% of the follicles—and/or PTGC-like features, defined by variable enlarged disrupted GC with interposed mantle zone B cells in at least one follicle.

For the T cell component, the CD4^+^/CD8^+^ ratio in the paracortical zone was given. This was considered normal between two and four, with a prevalence of CD4^+^ or CD8^+^ if it was five or more or one or less, respectively. Moreover, any increase in CD8^+^ cells into the GC, in PD1+ Tfh cells inside the GC, and in PD1+ cells in the paracortical zone was noted. Due to the absence of reliable cutoff values, this was defined descriptively as a significant rise compared to that expected in normal conditions. Any increase in paracortical TIA1+ T cells was recorded and defined as expanded if numbers were comparable to CD8^+^ T cells.

For the plasma cell component, the absolute number was counted in the interfollicular/medullary zone—identified by CD138 and/or MUM1 stain—counting the absolute median number in three hotspot high-power fields (HPFs), and any increase in plasma cells into the GC noted; again, due to the absence of reliable cutoffs, this was defined as an increase compared to that expected in normal conditions. For both the interfollicular and medullary plasma cells and plasma cells into the GC, the absolute number of IgG, IgM, and IgD-positive plasma cells was detected (absolute median number in three hotspot HPF). Additionally, the number of intra-GC IgG4+ plasma cells was counted (absolute median number in three hotspot HPF).

The presence of granulomas was recorded, as well as any increase in CD68^+^ or CD163+ interfollicular macrophages—defined descriptively by a significant rise compared to that expected in normal conditions.

The presence of aggregates of CD123+/CD303+ pDCs was considered (if less or more than 3 in absolute number), and an increase in CD30^+^ interfollicular immunoblasts was also examined; as before, this was defined descriptively by a significant increase compared to what might be expected in normal conditions.

Finally, the presence of EBV-positive cells was screened through in situ hybridization (EBER), with rare positive cells considered as a normal finding of carrier cells.

### Genetic characterization

Genetic analysis was performed on genomic DNA extracted from peripheral blood through Sanger sequencing or NGS techniques according to Table 1.

Coding exons and adjacent intronic junctions were analyzed by sequencing with the Ion Torrent S5 System Sequencer and Ion Reporter Software 5.18 (Thermo Fisher Scientific). Sequencing depth was 100× for 94% of the nucleotides; end-to-end coverages were at least 20× in 98.25% of the analyzed regions (90). Not all patients underwent the same genetic testing, as they were enrolled in two different centers.

15 out of 38 patients were tested with a WES.

21 out of 38 patients underwent a 49-gene panel for IEI typically associated with immune dysregulation, including the following genes: *AICDA*, *AIRE*, *ATM*, *BACH2*, *BTK*, *CARD11*, *CASP10*, *CASP8*, *CD19*, *CD40*, *CD40LG*, *CD79A*, *CD79B*, *CD81*, *CECR1*, *CR2*, *CTLA-4*, *DOCK8*, *FAS*, *FASLG*, *ICOS*, *IL2RA*, *IL2RB*, *IL6R*, *IL6ST*, *LRBA*, *MAGT1*, *MS4A1*, *NFKB1*, *NFKB2*, *NFKBIA*, *PIK3CD*, *PIK3R1*, *PLCG2*, *PRF1*, *PRKCD*, *RAG1*, *RAG2*, *SH2D1A*, *STAT1*, *STAT3*, *STX11*, *STXBP2*, *TNFRSF13B*, *TRNT1*, *TYK2*, *UNC13D*, *UNG*, and *XIAP*.

Two out of 38 patients were tested using a 525-gene panel for IEI, including the following genes: *ABCG5*, *ABCG8*, *ACD*, *ACP5*, *ACTB*, *ADA*, *ADA2*, *ADAM17*, *ADAR*, *AICDA*, *AIRE*, *AK1*, *AK2*, *ALAS2*, *ALDOA*, *ALG12*, *ANK1*, *AP1S3*, *AP3B1*, *AP3D1*, *APOL1*, *ARHGEF1*, *ARPC1B*, *ATM*, *ATP6AP1*, *ATP6AP2*, *B2M*, *BACH2*, *BANK1*, *BCL10*, *BCL11B*, *BLM*, *BLNK*, *BLOC1S3*, *BLOC1S6*, *BPGM*, *BRIP1*, *BTK*, *C1GALT1C1*, *C1QA*, *C1QB*, *C1QC*, *C1R*, *C1S*, *C2*, *C3*, *C4A*, *C5*, *C6*, *C7*, *C8A*, *C8B*, *C9*, *CARD11*, *CARD14*, *CARD9*, *CARMIL2*, *CASP10*, *CASP8*, *CAV1*, *CAVIN1*, *CCBE1*, *CD19*, *CD244*, *CD247*, *CD27*, *CD3D*, *CD3E*, *CD3G*, *CD40*, *CD40LG*, *CD46*, *CD55*, *CD59*, *CD70*, *CD79A*, *CD79B*, *CD81*, *CD8A*, *CDAN1*, *CDC42*, *CDCA7*, *CDIN1*, *CEBPE*, *CFB*, *CFD*, *CFH*, *CFHR1*, *CFHR2*, *CFHR3*, *CFHR4*, *CFHR5*, *CFI*, *CFP*, *CFTR*, *CHD7*, *CIB1*, *CIITA*, *CISD2*, *CLCN7*, *CLCNKB*, *CLEC7A*, *COG6*, *COL4A1*, *COLEC11*, *COMT*, *COPA*, *CORIN*, *CORO1A*, *CR2*, *CSF2RA*, *CSF2RB*, *CTC1*, *CTLA4*, *CTNNBL1*, *CTPS1*, *CTSC*, *CXCR4*, *CYB5R3*, *CYBA*, *CYBB*, *CYBC1*, *DBR1*, *DCLRE1B*, *DCLRE1C*, *DDX41*, *DEF6*, *DGAT1*, *DGKE*, *DKC1*, *DNAJC21*, *DNASE1*, *DNASE1L3*, *DNASE2*, *DNMT3B*, *DOCK2*, *DOCK8*, *DTNBP1*, *EFL1*, *ELANE*, *EPB41*, *EPB42*, *EPG5*, *ERCC2*, *ERCC3*, *ERCC4*, *ERCC6L2*, *EXTL3*, *FAAP24*, *FADD*, *FANCA*, *FANCB*, *FANCC*, *FANCD2*, *FANCE*, *FANCF*, *FANCG*, *FANCI*, *FANCL*, *FANCM*, *FAS*, *FASLG*, *FAT4*, *FCGR2A*, *FCGR2B*, *FCGR3A*, *FCHO1*, *FCN3*, *FERMT1*, *FERMT3*, *FLNA*, *FNIP1*, *FOXD3*, *FOXN1*, *FOXP3*, *FPR1*, *G6PC3*, *G6PD*, *GALC*, *GATA1*, *GATA2*, *GCLC*, *GFI1*, *GINS1*, *GPI*, *GSR*, *GSS*, *GYPC*, *HAVCR2*, *HAX1*, *HELLS*, *HK1*, *HMOX1*, *HNF1A*, *HPS1*, *HPS3*, *HPS4*, *HPS5*, *HPS6*, *HTRA2*, *HYOU1*, *ICOS*, *ICOSLG*, *IFIH1*, *IFNAR1*, *IFNAR2*, *IFNGR1*, *IFNGR2*, *IGHG2*, *IGHM*, *IGKC*, *IGLL1*, *IKBKB*, *IKZF1*, *IKZF3*, *IL10*, *IL10RA*, *IL10RB*, *IL12B*, *IL12RB1*, *IL12RB2*, *IL17F*, *IL17RA*, *IL17RC*, *IL18BP*, *IL1RN*, *IL21*, *IL21R*, *IL23R*, *IL2RA*, *IL2RB*, *IL2RG*, *IL36RN*, *IL6*, *IL6R*, *IL6ST*, *IL7R*, *INO80*, *IPO8*, *IRAK1*, *IRAK4*, *IRF2BP2*, *IRF3*, *IRF4*, *IRF7*, *IRF8*, *IRF9*, *ISG15*, *ITCH*, *ITGAM*, *ITGB2*, *ITK*, *ITPR3*, *IVNS1ABP*, *JAK1*, *JAK3*, *KCNN4*, *KDM6A*, *KIF23*, *KLF1*, *KMT2A*, *KMT2D*, *KRAS*, *LACC1*, *LAMTOR2*, *LAT*, *LCK*, *LIG1*, *LIG4*, *LMNB2*, *LPIN2*, *LRBA*, *LRRC8A*, *LYST*, *MAD2L2*, *MAGT1*, *MALT1*, *MAN2B1*, *MAPK8*, *MASP1*, *MASP2*, *MBL2*, *MCM4*, *MEFV*, *MIF*, *MMP2*, *MOGS*, *MPO*, *MRE11*, *MRTFA*, *MS4A1*, *MSH6*, *MSN*, *MTHFD1*, *MVK*, *MYD88*, *MYSM1*, *NBAS*, *NBN*, *NCF1*, *NCF2*, *NCF4*, *NCKAP1L*, *NCSTN*, *NEIL3*, *NFAT5*, *NFE2L2*, *NFKB1*, *NFKB2*, *NFKBIA*, *NFKBIL1*, *NHEJ1*, *NHP2*, *NKX2-5*, *NLRC4*, *NLRP1*, *NLRP12*, *NLRP2*, *NLRP3*, *NOD2*, *NOP10*, *NRAS*, *NSMCE3*, *NT5C3A*, *OAS1*, *ORAI1*, *OSM*, *OSTM1*, *OTULIN*, *PADI4*, *PALB2*, *PARN*, *PAX4*, *PAX5*, *PEPD*, *PFKM*, *PGK1*, *PGM3*, *PIEZO1*, *PIGA*, *PIK3CD*, *PIK3CG*, *PIK3R1*, *PKLR*, *PLCG2*, *PLEKHM1*, *PMM2*, *PMS2* (exons 1–10), *PNP*, *POLA1*, *POLD1*, *POLD2*, *POLE*, *POLE2*, *POLG*, *POLR3A*, *POLR3F*, *PRF1*, *PRKCD*, *PRKDC*, *PSEN1*, *PSENEN*, *PSMB8*, *PSMG2*, *PSTPIP1*, *PTEN*, *PTPN22*, *PTPRC*, *PTPRT*, *RAB27A*, *RAC2*, *RAD51*, *RAD51C*, *RAG1*, *RAG2*, *RANBP2*, *RASGRP1*, *RBCK1*, *RBM8A*, *RC3H1*, *REL*, *RELA*, *RELB*, *RFWD3*, *RFX5*, *RFXANK*, *RFXAP*, *RHAG*, *RHOH*, *RIGI*, *RIPK1*, *RMRP*, *RNASEH2A*, *RNASEH2B*, *RNASEH2C*, *RNF113A*, *RNF168*, *RNF31*, *RNU4ATAC*, *RORC*, *RPA1*, *RPSA*, *RTEL1*, *SAMD9*, *SAMD9L*, *SAMHD1*, *SASH3*, *SBDS*, *SEC23B*, *SEC61A1*, *SEMA3E*, *SEMA6B*, *SERPING1*, *SH2D1A*, *SH3BP2*, *SH3KBP1*, *SKIC2*, *SKIC3*, *SLC12A3*, *SLC22A4*, *SLC29A3*, *SLC2A1*, *SLC35C1*, *SLC37A4*, *SLC39A7*, *SLC46A1*, *SLC4A1*, *SLC5A6*, *SLC7A7*, *SLX4*, *SMARCAL1*, *SMG6*, *SMIM1*, *SMPD1*, *SNX10*, *SOCS1*, *SP110*, *SPI1*, *SPINK5*, *SPPL2A*, *SPTA1*, *SPTB*, *SRP72*, *STAT1*, *STAT2*, *STAT3*, *STAT4*, *STAT5B*, *STAT6*, *STIM1*, *STING1*, *STK4*, *STN1*, *STOX1*, *STX11*, *STXBP2*, *SYK*, *TAFAZZIN*, *TAP1*, *TAP2*, *TAPBP*, *TBK1*, *TBX1*, *TBX2*, *TBX21*, *TCF3*, *TCIRG1*, *TCN2*, *TERC*, *TERT*, *TET2*, *TFRC*, *TGFB1*, *TGFBR1*, *TGFBR2*, *THBD*, *TICAM1*, *TINF2*, *TIRAP*, *TLR3*, *TLR7*, *TLR8*, *TMC6*, *TMC8*, *TNFAIP3*, *TNFRSF11A*, *TNFRSF13B*, *TNFRSF13C*, *TNFRSF1A*, *TNFRSF4*, *TNFRSF9*, *TNFSF11*, *TNFSF12*, *TONSL*, *TOP2B*, *TP53*, *TPI1*, *TPP1*, *TPP2*, *TRAC*, *TRAF3*, *TRAF3IP2*, *TREX1*, *TRIM22*, *TRNT1*, *TTC7A*, *TYK2*, *UBE2T*, *UNC119*, *UNC13D*, *UNC93B1*, *UNG*, *USB1*, *USP18*, *VPS13B*, *VPS45*, *WAS*, *WDR1*, *WIPF1*, *WRAP53*, *XIAP*, *XRCC2*, *ZAP70*, *ZBTB24*, and *ZNF341*.

To interpret the results, the following public databases were consulted: VarSome (https://varsome.com), ClinVar (https://www.ncbi.nlm.nih.gov/clinvar/), and Infevers (https://infevers.umai-montpellier.fr/web/index.php).

### Statistical analysis

Descriptive statistics included the frequency as appropriate for categorical variables and the mean (95% confidence interval) for continuous variables. All analyses were performed using Prism software (GraphPad Software) and Microsoft Excel version 2013. PCA was performed on the frequencies of T and B cell subsets using PAST software (PAleontological STatistics, version 3.22; University of Oslo, Oslo, Norway) to visualize data structure and assess correlations among immunophenotypic variables (91).

### Statistical methods

The analysis was designed to evaluate the statistical significance of differences between groups, defined by the presence or absence of a categorical variable. Differences in frequency were tested using Χ^2^ tests, while mean differences were assessed using Student’s *t* tests, with a two-tailed P value threshold of <0.05 for significance. Additionally, the study examined correlations within groups using the Pearson correlation coefficient, considering a two-tailed P value of <0.05 to indicate significant correlation.

To account for the multiple immunophenotypic comparisons performed, we applied two complementary procedures for multiple-testing correction: the conservative Bonferroni correction and the false discovery rate procedure by BH. For each statistically significant association, we report the raw P value and note whether the result remained significant after Bonferroni and BH adjustment.

### Online supplemental material

The manuscript references several supplementary tables and figures required to fully elucidate the clinical and histological data. Table S1 provides a comprehensive breakdown of clinical features and specific therapeutic management for the cohort. Tables S2, S4, and S5 contain statistical comparisons between patients with overt lymphoid neoplasms versus nonneoplastic/reactive LPD, covering clinical parameters, and quantitative and qualitative immunologic features. Table S3 offers a heatmap of raw immunological variables for each individual patient. Table S6 provides salient clues for pathologists evaluating LPD in the suspicion of an underlying IEI. Finally, the supplementary figures include Figs. S1 and S2, which provide representative histological images of findings such as follicular hyperplasia and specific immunostainings (CD8^+^, PD1+, and IRF4+), while Figs. S3 and S4 present sunburst diagrams categorizing patients by IEI classification and specific LPD diagnoses.

## Ethics statement

This work has been carried out in accordance with The Code of Ethics of the World Medical Association (Declaration of Helsinki) for experiments involving humans. All participants and/or their legal guardians provided informed written consent before their enrollment in this study.

## Supplementary Material

Table S1 shows the overall clinical features and therapeutic management of the cohort.

Table S2 shows the comparison of clinical parameters between overt lymphoid neoplasm (lymphoma) and nonneoplastic/reactive LPD groups.

Table S3 shows the heatmap of immunological features of the cohort.

Table S4 shows the comparison of quantitative immunologic parameters between overt lymphoid neoplasm (lymphoma) and nonneoplastic/reactive LPD groups.

Table S5 shows the comparison of qualitative immunologic parameters between overt lymphoid neoplasm (lymphoma) and nonneoplastic/reactive LPD groups.

Table S6 shows the salient clues for pathologists in the suspicion of an underlying IEI.

## Data Availability

Data are available in the article itself and its supplementary materials. All clinical, genetic, histopathological, and immunological data supporting the findings of this study are fully included within the published manuscript and its accompanying supplementary files. No additional datasets were generated or analyzed beyond those presented. Further clarifications or methodological details may be provided upon reasonable request to the corresponding authors.
