# Supplementary material for: Lymphoproliferation in inborn errors of immunity: From challenging diagnosis to histologic revision
Source: J Hum Immun. 2026 Feb 13;2(2):e20250174. doi: 10.70962/jhi.20250174 (PMC13177383; doi:10.70962/jhi.20250174)
Supplement: Table S2 — shows the comparison of clinical parameters between overt lymphoid neoplasm (lymphoma) and nonneoplastic/reactive LPD groups. [file jhi_20250174_tables2.docx]

**Table S2.** Comparison of clinical parameters between overt lymphoid neoplasm (lymphoma) and non-neoplastic/reactive LPD groups

| **Variables** | | **Malignant LPD group (n=9)** | **Non-malignant LPD group (n=29)** | **Test** |
| --- | --- | --- | --- | --- |
| *Quantitative variables*  *(T test)* |  | **Mean (SD)** | **Mean (SD)** | **T test** |
|  | Age at clinical onset (years) (SD) | 4.35 (4.40) | 12.06 (13.29) | **0.012*  ***1.788*  ****0.596* |
|  | Age at signs leading to IEI diagnosis (years) (SD) | 11.89 (10.94) | 16.81 (16.21) | 0.281 |
|  | Age at clinical IEI diagnosis (years) (SD) | 13.05 (11.74) | 19.13 (17.15) | 0.212 |
|  | Age at genetic IEI diagnosis (years) (SD) | 14.00 (11.88) | 17.80 (11.60) | 0.545 |
|  | Age at last follow-up (years) (SD) | 22.58 (13.94) | 25.23 (15.76) | 0.607 |
|  | Diagnostic delay from first signs (years) (SD) | 8.69 (9.75) | 7.08 (7.38) | 0.616 |
|  | Diagnostic delay from signs leading to diagnosis (years) (SD) | 1.16 (1.79) | 2.33 (2.77) | 0.129 |
|  | Time from clinical to genetic IEI diagnosis (years) (SD) | 5.33 (7.61) | 2.50 (4.35) | 0.432 |
|  | Age at serological evaluation (years) (SD) | 15.17 (13.20) | 21.80 (15.36) | 0.184 |
|  |  | **Observations** | **Observations** | ***Chi-squared / Fisher*** |
| *Qualitative variables*  *(Chi-squared / Fisher)* | Sex (female) | 4/9 | 12/29 | 0.970 |
|  | Autoimmunity as first clinical signs | 1/9 | 12/29 | 0.486 |
|  | LPDs as first clinical signs | 5/9 | 10/29 | 0.367 |
|  | Infections as first clinical signs | 6/9 | 15/29 | 0.486 |
|  | Autoimmunity as signs leading to IEI diagnosis | 1/9 | 12/29 | 0.158 |
|  | LPDs as signs leading to IEI diagnosis | 7/9 | 23/29 | 0.232 |
|  | Infections as signs leading to IEI diagnosis | 5/9 | 18/29 | 0.850 |
|  | Autoimmunity during follow-up | 3/9 | 16/29 | 0.485 |
|  | Infections during follow-up | 2/9 | 9/29 | 0.714 |
|  | Autoimmunity at last follow-up | 1/9 | 13/29 | 0.472 |
|  | LPDs at last follow-up | 9/9 | 29/29 | 1.000 |
|  | Infections at last follow-up | 6/9 | 18/29 | 0.675 |
|  | Pulmonary immune dysregulation | 3/9 | 16/29 | 0.079 |
|  | Gastro-intestinal involvement | 2/9 | 10/29 | 0.714 |
|  | Splenomegaly | 6/9 | 23/29 | 1.000 |
|  | Liver involvement | 1/9 | 10/29 | 0.444 |
|  | Endocrinopathy | 2/9 | 7/29 | 0.689 |
|  | Dermatologic involvement | 2/9 | 11/29 | 0.486 |
|  | Rheumatologic manifestations | 0/9 | 6/29 | 1.000 |
|  | Hematologic manifestations | 9/9 | 29/29 | 1.000 |

***Table 2S.*** *Abbreviations: IEI, inborn error of immunity; LPD, lymphoid proliferation; SD, standard deviation.*

** Statistically significant*

*** Bonferroni correction*

**** P-value adjusted as per Benjamini-Hochberg method*
