# Supplementary material for: Lymphoproliferation in inborn errors of immunity: From challenging diagnosis to histologic revision
Source: J Hum Immun. 2026 Feb 13;2(2):e20250174. doi: 10.70962/jhi.20250174 (PMC13177383; doi:10.70962/jhi.20250174)
Supplement: Table S3 — shows the heatmap of immunological features of the cohort. [file jhi_20250174_tables3.docx]

**Table S3.** Heatmap of immunological features of the cohort

|  | | NON-GENETICALLY DEFINED CID(-LIKE) | | | | | | | | ATM | | | CVID/CVID-LIKE | | | | | | | | | | | | | | | APDS | | | | | CTLA4 DEFICIENCY | | ALPS | | ALPS-LIKE | sALPS | XMEN |
| --- | --- | --- | --- | --- | --- | --- | --- | --- | --- | --- | --- | --- | --- | --- | --- | --- | --- | --- | --- | --- | --- | --- | --- | --- | --- | --- | --- | --- | --- | --- | --- | --- | --- | --- | --- | --- | --- | --- | --- |
| **Immunological variables** | | P1 | P2 | P3 | P4 | P5 | P6 | P7 | P8 | P9 | P10 | P11 | P12 | P13 | P14 | P15 | P16 | P17 | P18 | P19 | P20 | P21 | P22 | P23 | P24 | P25 | P26 | P27 | P28 | P29 | P30 | P31 | P32 | P33 | P34 | P35 | P36 | P37 | P38 |
| Basic features | Hemoglobin (g/dl) |  |  |  |  |  |  |  |  |  |  |  |  |  |  |  |  |  |  |  |  |  |  |  |  |  |  |  |  |  |  |  |  |  |  |  |  |  |  |
|  | Platelets (cell/ul) |  |  |  |  |  |  |  |  |  |  |  |  |  |  |  |  |  |  |  |  |  |  |  |  |  |  |  |  |  |  |  |  |  |  |  |  |  |  |
|  | WBC (cell/ul) |  |  |  |  |  |  |  |  |  |  |  |  |  |  |  |  |  |  |  |  |  |  |  |  |  |  |  |  |  |  |  |  |  |  |  |  |  |  |
|  | Neutrophils (cell/ul) |  |  |  |  |  |  |  |  |  |  |  |  |  |  |  |  |  |  |  |  |  |  |  |  |  |  |  |  |  |  |  |  |  |  |  |  |  |  |
|  | Eosinophils (cell/ul) |  |  |  |  |  | NA |  |  |  |  |  |  |  |  |  |  | NA |  |  |  |  |  |  |  |  |  |  |  |  |  |  |  |  |  |  |  |  |  |
|  | Lymphocytes (cell/ul) |  |  |  |  |  |  |  |  |  |  |  |  |  |  |  |  |  |  |  |  |  |  |  |  |  |  |  |  |  |  |  |  |  |  |  |  |  |  |
|  | CD3+ PAN-T cells (%‡) |  |  |  |  |  |  |  |  |  |  |  |  |  |  |  |  |  |  |  |  |  |  |  |  |  |  |  |  |  |  |  |  |  |  |  |  |  |  |
| CD4+ T-cell subsets | CD3+CD4+ T cells (cell/ul) |  |  |  |  |  |  |  |  |  |  |  |  |  |  |  |  |  |  |  |  |  |  |  |  |  |  |  |  |  |  |  |  |  |  |  |  |  |  |
|  | CD3+CD4+ T cells (%‡) |  |  |  |  |  |  |  |  |  |  |  |  |  |  |  |  |  |  |  |  |  |  |  |  |  |  |  |  |  |  |  |  |  |  |  |  |  |  |
|  | CD4+CD45RA+CD27+ naїve T cells (%§) |  |  |  |  |  |  |  |  |  |  |  |  |  |  |  |  |  |  |  |  |  |  |  |  |  |  |  |  |  |  |  |  |  |  |  |  |  |  |
|  | CD4+CD45RA-CD27+ central memory T cells (%§) |  |  |  |  |  |  |  |  |  |  |  |  |  |  |  |  |  |  |  |  |  |  |  |  |  |  |  |  |  |  |  |  |  |  |  |  |  |  |
|  | CD4+CD45RA-CD27- effector memory T cells (%§) |  |  |  |  |  |  |  |  |  |  |  |  |  |  |  |  |  |  |  |  |  |  |  |  |  |  |  |  |  |  |  |  |  |  |  |  |  |  |
|  | CD4+CD45RA+CD27- terminal effector memory T cells (%§) |  |  |  |  |  |  |  |  |  |  |  |  |  |  |  |  | NA |  |  |  |  |  |  |  |  |  |  |  |  |  |  |  |  |  |  |  |  |  |
|  | CD4+CD127-CD27+CD25++ regulatory T cells (%§) | NA | NA | NA | NA | NA |  |  | NA |  | NA | NA |  |  |  |  |  |  |  |  |  |  | NA | NA |  | NA | NA |  |  | NA | NA | NA |  | NA |  |  |  | NA | NA |
|  | CD4+CD45RA-CXCR5+ follicular helper T cells (% CD4+CD45RA-) |  |  |  |  |  | NA | NA |  | NA | NA |  | NA | NA | NA | NA | NA | NA | NA | NA | NA | NA |  |  |  |  |  | NA |  |  |  |  | NA |  | NA | NA | NA |  | NA |
| CD8+ T-cell subsets | CD3+CD8+ T cells (cell/ul) |  |  |  |  |  |  |  |  |  |  |  |  |  |  |  |  |  |  |  |  |  |  |  |  |  |  |  |  |  |  |  |  |  |  |  |  |  |  |
|  | CD3+CD8+ T cells (%‡) |  |  |  |  |  |  |  |  |  |  |  |  |  |  |  |  |  |  |  |  |  |  |  |  |  |  |  |  |  |  |  |  |  |  |  |  |  |  |
|  | CD8+CD45RA+CCR7+ naїve T cells (%¶) |  |  |  |  |  |  |  |  |  |  |  |  |  |  |  |  |  |  |  |  |  |  |  |  |  |  |  |  |  |  |  |  |  |  |  |  |  |  |
|  | CD8+CD45RA-CCR7+ central memory T cells (%¶) |  |  |  |  |  |  |  |  |  |  |  |  |  |  |  |  |  |  |  |  |  |  |  |  |  |  |  |  |  |  |  |  |  |  |  |  |  |  |
|  | CD8+CD45RA-CCR7- effector memory T cells (%¶) |  |  |  |  |  |  |  |  |  |  |  |  |  |  |  |  |  |  |  |  |  |  |  |  |  |  |  |  |  |  |  |  |  |  |  |  |  |  |
|  | CD8+CD45RA+CCR7- late effector T cells (%¶) |  |  |  |  |  |  |  |  |  |  |  |  |  |  |  |  |  |  |  |  |  |  |  |  |  |  |  |  |  |  |  |  |  |  |  |  |  |  |
| Other cell subsets | CD56+CD16+CD3- natural killer cells (cell/ul) |  |  |  |  |  |  |  |  |  |  |  |  |  |  |  |  |  |  |  |  |  |  |  |  |  |  |  |  |  |  |  |  |  |  |  |  |  |  |
|  | CD56+CD16+CD3- natural killer cells (%‡) |  |  |  |  |  |  |  |  |  |  |  |  |  |  |  |  |  |  |  |  |  |  |  |  |  |  |  |  |  |  |  |  |  |  |  |  |  |  |
|  | TCRαβ+CD3+CD4-CD8- double negative T cells (%††) |  |  |  |  |  |  |  |  |  | NA |  |  |  |  |  |  | NA |  |  |  |  |  |  |  |  |  |  |  |  |  |  |  |  |  |  |  |  |  |
|  | CD3+γ+δ+ (%‡) |  |  |  |  |  |  |  |  |  | NA |  |  |  |  |  |  |  |  |  |  |  |  |  |  |  |  |  |  |  |  |  |  |  |  |  |  |  |  |
| CD19+ B-cell subsets | CD19+ PAN-B cells (cell/ul) |  |  |  |  |  |  |  |  |  |  |  |  |  |  |  |  |  |  |  |  |  |  |  |  |  |  |  |  |  |  |  |  |  |  |  |  |  |  |
|  | CD19+ PAN-B cells (%‡) |  |  |  |  |  |  |  |  |  |  |  |  |  |  |  |  |  |  |  |  |  |  |  |  |  |  |  |  |  |  |  |  |  |  |  |  |  |  |
|  | CD19+IgD+CD27- naïve B cells (%‡‡) |  |  |  | NA |  |  | NA |  |  |  |  |  |  |  |  |  |  |  |  |  |  |  |  |  |  |  |  |  |  |  |  | NA |  |  |  |  |  |  |
|  | CD19+IgM++CD38++ transitional B cells (%‡‡) |  |  |  | NA |  |  | NA |  |  |  |  | NA |  |  |  |  | NA |  |  |  |  |  |  |  |  |  |  |  |  |  |  | NA |  |  |  |  |  |  |
|  | CD19+IgD+CD27+ memory B cells (%‡‡) |  |  |  | NA |  |  | NA |  |  |  |  |  |  |  |  |  |  |  |  |  |  |  |  |  |  |  |  |  |  |  |  | NA |  |  |  |  |  |  |
|  | CD19+IgD-CD27+ switched memory B cells (%‡‡) |  |  |  | NA |  |  | NA |  |  |  |  |  |  |  |  |  |  |  |  |  |  |  |  |  |  |  |  |  |  |  |  | NA |  |  |  |  |  |  |
|  | CD19+CD21+lCD38- CD21low B cells (%‡‡) |  |  |  | NA |  |  | NA |  |  |  |  | NA |  |  |  |  | NA |  |  |  |  |  |  |  |  |  |  |  |  |  |  | NA |  |  |  |  |  |  |
|  | CD19+IgM-+CD38++ plasmablasts (%‡‡) |  |  |  | NA |  |  | NA |  |  |  |  | NA |  |  |  |  | NA |  |  |  |  |  |  |  |  |  |  |  |  |  | NA | NA |  |  |  |  |  |  |
| Immunoglobulins | IgG (mg/dl)§§ |  |  |  |  |  |  |  |  |  |  |  |  |  |  |  |  |  |  |  |  |  |  |  |  |  |  |  |  |  |  |  |  |  |  |  |  |  |  |
|  | IgA (mg/dl)§§ |  |  |  |  |  |  |  |  |  |  |  |  |  |  |  |  |  |  |  |  |  |  |  |  |  |  |  |  |  |  |  |  |  |  |  |  |  |  |
|  | IgM (mg/dl)§§ |  |  |  |  |  |  |  |  |  |  |  |  |  |  |  |  |  |  |  |  |  |  |  |  |  |  |  |  |  |  |  |  |  |  |  |  |  |  |
|  | IgE (UI/ml) |  |  |  |  |  |  |  |  |  | NA |  |  |  |  |  |  |  |  |  |  |  | NA |  |  |  |  |  |  |  |  |  |  |  |  |  |  | NA | NA |
|  | IgG1 (mg/dl)§§ |  | NA |  | NA |  |  |  |  | NA |  | NA |  |  |  |  |  | NA | NA |  |  | NA | NA | NA |  |  | NA |  | NA | NA |  | NA |  | NA | NA |  |  | NA | NA |
|  | IgG2 (mg/dl)§§ |  | NA |  | NA |  |  |  |  | NA |  | NA |  |  |  |  |  | NA | NA |  |  | NA | NA | NA |  |  | NA |  | NA | NA |  | NA |  | NA | NA |  |  | NA | NA |
|  | IgG3 (mg/dl)§§ |  | NA |  | NA |  |  |  |  | NA |  | NA |  |  |  |  |  | NA | NA |  |  | NA | NA | NA |  |  | NA |  | NA | NA |  | NA |  | NA | NA |  |  | NA | NA |
|  | IgG4 (mg/dl)§§ |  | NA |  | NA |  |  |  |  | NA |  | NA |  |  |  |  |  | NA | NA |  |  | NA | NA | NA |  |  | NA |  | NA | NA |  | NA |  | NA | NA |  |  | NA | NA |
|  |  | P1 | P2 | P3 | P4 | P5 | P6 | P7 | P8 | P9 | P10 | P11 | P12 | P13 | P14 | P15 | P16 | P17 | P18 | P19 | P20 | P21 | P22 | P23 | P24 | P25 | P26 | P27 | P28 | P29 | P30 | P31 | P32 | P33 | P34 | P35 | P36 | P37 | P38 |
|  |  | NON-GENETICALLY DEFINED CID(-LIKE) | | | | | | | | ATM | | | CVID/CVID-LIKE | | | | | | | | | | | | | | | APDS | | | | | CTLA4 DEFICIENCY | | ALPS | | ALPS-LIKE | sALPS | XMEN |

***Table 3S****. Abbreviations: ALPS, autoimmune lymphoproliferative syndrome; APDS, activated PI3K-delta syndrome; ATM, ataxia-telangiectasia; CID, combined immune deficiency; CTLA4, Cytotoxic T-lymphocyte-associated protein 4; CVID, common variable immune deficiency; IEI, inborn error of immunity; NA, not assessed; sALPS, somatic ALPS; WBC, white blood cells; XMEN, X-linked immunodeficiency with magnesium defect.*

*Legend:* *Values over the upper limit of normal for age are reported in red, values under the lower limit of normal in blue, values in range in grey.*

*† % total WBC.*

*‡ % total lymphocytes.*

*§ % total CD4+ cells.*

*¶ % total CD8+ cells.*

*†† % TCRαβ+CD3+ cells.*

*‡‡ % total CD19+ cells*

*§§ SI conversion factor: To convert IgG/IgA/IgM to g/L, multiply values by 10².*
