# Supplementary material for: Lymphoproliferation in inborn errors of immunity: From challenging diagnosis to histologic revision
Source: J Hum Immun. 2026 Feb 13;2(2):e20250174. doi: 10.70962/jhi.20250174 (PMC13177383; doi:10.70962/jhi.20250174)
Supplement: Table S4 — shows the comparison of quantitative immunologic parameters between overt lymphoid neoplasm (lymphoma) and nonneoplastic/reactive LPD groups. [file jhi_20250174_tables4.docx]

**Table S4.** Comparison of quantitative immunologic parameters between overt lymphoid neoplasm (lymphoma) and non-neoplastic/reactive LPD groups

| **Variables** | | **Malignant LPD group (n=9)** | **Non-malignant LPD group (n=29)** | **T-test** |
| --- | --- | --- | --- | --- |
|  |  | **Mean [SD]** | **Mean [SD]** | **P-value** |
| *Basic features* | Hemoglobin (g/dl) | 13.11 [1.38] | 13.56 [1.89] | 0.416 |
|  | Platelets (cell/ul) | 248417.00 [105692.90] | 207808.00 [117331.70] | 0.299 |
|  | WBC (cell/ul) | 6097.50 [2452.10] | 6105.77 [2257.02] | 0.992 |
|  | Neutrophils (cell/ul) | 3548.33 [2096.58] | 3632.69 [1458.25] | 0.901 |
|  | Eosinophils (cell/ul) | 646.36 [941.84] | 187.20 [270.15] | 0.141 |
|  | Lymphocytes (cell/ul) | 1757.50 [696.45] | 1937.69 [1205.75] | 0.565 |
|  | CD3+ PAN-T cells (%‡) | 68.15 [16.31] | 78.02 [9.81] | 0.072 |
| *CD4+ T-cell*  *subsets* | CD3+CD4+ T cells (cell/ul) | 560.31 [271.26] | 765.01 [510.06] | 0.116 |
|  | CD3+CD4+ T cells (%‡) | 32.46 [10.83] | 39.71 [10.82] | 0.068 |
|  | CD4+CD45RA+CD27+ naїve T cells (%§) | 30.91 [28.96] | 28.67 [17.27] | 0.808 |
|  | CD4+CD45RA-CD27+ central memory T cells (%§) | 46.58 [22.31] | 52.60 [15.08] | 0.408 |
|  | CD4+CD45RA-CD27- effector memory T cells (%§) | 22.07 [25.99] | 16.74 [11.12] | 0.507 |
|  | CD4+CD45RA+CD27- terminal effector memory T cells (%§) | 2.70 [2.46] | 2.15 [4.08] | 0.630 |
|  | CD4+CD127-CD27+CD25++ regulatory T cells (%§) | 4.42 [3.84] | 4.31 [2.50] | 0.930 |
|  | CD4+CD45RA-CXCR5+ follicular helper T cells (%§) | 9.92 [2.56] | 18.08 [11.88] | **0.036*  ***5.364*  ****1.000* |
| *CD8+ T-cell*  *subsets* | CD3+CD8+ T cells (cell/ul) | 543.97 [367.61] | 599.79 [474.55] | 0.696 |
|  | CD3+CD8+ T cells (%‡) | 30.57 [14.52] | 30.96 [10.01] | 0.934 |
|  | CD8+CD45RA+CCR7+ naїve T cells (%*¶*) | 20.03 [20.79] | 23.77 [18.06] | 0.598 |
|  | CD8+CD45RA-CCR7+ central memory T cells (%*¶*) | 6.41 [4.90] | 6.82 [12.34] | 0.884 |
|  | CD8+CD45RA-CCR7- effector memory T cells (%*¶*) | 25.66 [14.41] | 33.07 [15.30] | 0.162 |
|  | CD8+CD45RA+CCR7- late effector T cells (%*¶*) | 48.93 [23.03] | 36.83 [22.47] | 0.144 |
| *Other cell subsets* | CD56+CD16+CD3- natural killer cells (cell/ul) | 213.11 [139.80] | 148.45 [120.46] | 0.183 |
|  | CD56+CD16+CD3- natural killer cells (%‡) | 12.67 [9.49] | 8.47 [5.64] | 0.177 |
|  | TCRαβ+CD3+CD4-CD8- double negative T cells (%††) | 1.89 [1.31] | 2.48 [2.29] | 0.339 |
|  | CD3+γ+δ+ (%‡) | 5.58 [4.07] | 5.72 [6.20] | 0.935 |
| *CD19+ B-cell*  *subsets* | CD19+ PAN-B cells (cell/ul) | 330.54 [361.46] | 187.84 [184.79] | 0.218 |
|  | CD19+ PAN-B cells (%‡) | 16.41 [15.63] | 10.27 [7.87] | 0.219 |
|  | CD19+IgD+CD27- naïve B cells (%‡‡) | 85.71 [15.47] | 84.66 [15.91] | 0.852 |
|  | CD19+IgM++CD38++ transitional B cells (%‡‡) | 12.00 [15.14] | 16.11 [21.87] | 0.534 |
|  | CD19+IgD+CD27+ memory B cells (%‡‡) | 10.38 [9.15] | 10.62 [12.18] | 0.950 |
|  | CD19+IgD-CD27+ switched memory B cells (%‡‡) | 3.34 [3.80] | 2.99 [3.27] | 0.804 |
|  | CD19+CD21+lCD38- CD21low B cells (%‡‡) | 9.59 [11.34] | 9.77 [11.89] | 0.966 |
|  | CD19+IgM-+CD38++ plasmablasts (%‡‡) | 0.69 [1.64] | 0.75 [1.79] | 0.934 |
| *Immunoglobulin*  *levels* | IgG (mg/dl)§§ | 638.33 [225.97] | 674.08 [828.58] | 0.839 |
|  | IgA (mg/dl)§§ | 35.08 [42.02] | 102.93 [168.46] | 0.092 |
|  | IgM (mg/dl)§§ | 164.80 [225.40] | 88.41 [169.62] | 0.350 |
|  | IgE (mg/dl)§§ | 29.47 [92.93] | 42.83 [58.54] | 0.805 |
|  | IgG1 (mg/dl)§§ | 343.04 [84.09] | 360.11 [405.50] | 0.876 |
|  | IgG2 (mg/dl)§§ | 108.32 [80.98] | 151.90 [140.29] | 0.404 |
|  | IgG3 (mg/dl)§§ | 41.88 [34.61] | 50.06 [49.98] | 0.690 |
|  | IgG4 (mg/dl)§§ | 36.71 [59.07] | 9.30 [13.54] | 0.423 |

**Table 4S.** *Abbreviations: SD, standard deviation; WBC, white blood cells.*

** Statistically significant*

*** Bonferroni correction*

**** P-value adjusted as per Benjamini-Hochberg method*

*† % total WBC*

*‡ % total lymphocytes*

*§ % total CD4+ cells ¶ % total CD8+ cells*

*†† % TCRαβ+CD3+ cells*

*‡‡ % total CD19+ cells*

*§§ SI conversion factor: To convert IgG/IgA/IgM to g/L, multiply values by 10²*
