# Supplementary material for: Lymphoproliferation in inborn errors of immunity: From challenging diagnosis to histologic revision
Source: J Hum Immun. 2026 Feb 13;2(2):e20250174. doi: 10.70962/jhi.20250174 (PMC13177383; doi:10.70962/jhi.20250174)
Supplement: Table S5 — shows the comparison of qualitative immunologic parameters between overt lymphoid neoplasm (lymphoma) and nonneoplastic/reactive LPD groups. [file jhi_20250174_tables5.docx]

**Table S5.** Comparison of qualitative immunologic parameters between overt lymphoid neoplasm (lymphoma) and non-neoplastic/reactive LPD groups

| **Variables** | | **Malignant LPD group (n=12)** | **Non-malignant LPD group (n=26)** | **Chi2 /**  **Fisher** |
| --- | --- | --- | --- | --- |
|  |  | **Observations** | **Observations** | **P-value** |
| *Altered basic features* | 🡻Hemoglobin (g/dl) | 0/9 | 3/29 | 0.538 |
|  | 🡹Hemoglobin (g/dl) | 0/9 | 0/29 | 1.000 |
|  | 🡻Platelets (cell/ul) | 1/9 | 6/29 | 1.000 |
|  | 🡹Platelets (cell/ul) | 1/9 | 1/29 | 0.538 |
|  | 🡻WBC (cell/ul) | 2/9 | 5/9 | 0.176 |
|  | 🡹WBC (cell/ul) | 0/9 | 0/29 | 1.000 |
|  | 🡻Neutrophils (cell/ul) | 1/9 | 4/29 | 1.000 |
|  | 🡹Neutrophils (cell/ul) | 0/29 | 0/29 | 1.000 |
|  | 🡻Eosinophils (cell/ul) | 1/8 | 6/28 | 0.400 |
|  | 🡹Eosinophils (cell/ul) | 1/8 | 1/28 | 0.524 |
|  | 🡻Lymphocytes (cell/ul) | 1/9 | 8/29 | 1.000 |
|  | 🡹Lymphocytes (cell/ul) | 0/9 | 0/29 | 1.000 |
|  | 🡻CD3+ PAN-T cells (%‡) | 2/9 | 0/29 | 0.094 |
|  | 🡹CD3+ PAN-T cells (%‡) | 0/9 | 2/29 | 1.000 |
| *Altered CD4+ T-*  *cell subsets* | 🡻CD3+CD4+ T cells (cell/ul) | 3/9 | 11/29 | 0.296 |
|  | 🡹CD3+CD4+ T cells (cell/ul) | 0/9 | 0/29 | 1.000 |
|  | 🡻CD3+CD4+ T cells (%‡) | 3/9 | 1/29 | 0.084 |
|  | 🡹CD3+CD4+ T cells (%‡) | 0/9 | 1/29 | 1.000 |
|  | 🡻CD4+CD45RA+CD27+ naїve T cells (%§) | 5/9 | 14/29 | 0.728 |
|  | 🡹CD4+CD45RA+CD27+ naїve T cells (%§) | 0/9 | 0/29 | 1.000 |
|  | 🡻CD4+CD45RA-CD27+ central memory T cells (%§) | 0/9 | 0/29 | 1.000 |
|  | 🡹CD4+CD45RA-CD27+ central memory T cells (%§) | 1/29 | 0/29 | 0.316 |
|  | 🡻CD4+CD45RA-CD27- effector memory T cells (%§) | 0/9 | 0/29 | 1.000 |
|  | 🡹CD4+CD45RA-CD27- effector memory T cells (%§) | 2/9 | 9/29 | 0.714 |
|  | 🡻CD4+CD45RA+CD27- terminal effector memory T cells (%§) | 0/8 | 0/29 | 1.000 |
|  | 🡹CD4+CD45RA+CD27- terminal effector memory T cells (%§) | 1/8 | 2/29 | 1.000 |
|  | 🡻CD4+CD127-CD27+CD25++ regulatory T cells (%§) | 1/4 | 7/16 | 0.642 |
|  | 🡹CD4+CD127-CD27+CD25++ regulatory T cells (%§) | 0/4 | 0/16 | 1.000 |
| *Altered CD8+ T-*  *cell subsets* | 🡻CD3+CD8+ T cells (cell/ul) | 1/9 | 3/29 | 1.000 |
|  | 🡹CD3+CD8+ T cells (cell/ul) | 0/9 | 1/29 | 1.000 |
|  | 🡻CD3+CD8+ T cells (%‡) | 1/9 | 0/29 | 0.316 |
|  | 🡹CD3+CD8+ T cells (%‡) | 2/9 | 6/29 | 0.689 |
|  | 🡻CD8+CD45RA+CCR7+ naїve T cells (%*¶*) | 5/9 | 10/29 | 0.481 |
|  | 🡹CD8+CD45RA+CCR7+ naїve T cells (%*¶*) | 0/9 | 0/29 | 1.000 |
|  | 🡻CD8+CD45RA-CCR7+ central memory T cells (%*¶*) | 2/9 | 6/29 | 1.000 |
|  | 🡹CD8+CD45RA-CCR7+ central memory T cells (%*¶*) | 3/9 | 3/29 | 0.357 |
|  | 🡻CD8+CD45RA-CCR7- effector memory T cells (%*¶*) | 1/9 | 1/29 | 0.538 |
|  | 🡹CD8+CD45RA-CCR7- effector memory T cells (%*¶*) | 0/9 | 0/29 | 1.000 |
|  | 🡻CD8+CD45RA+CCR7- late effector T cells (%*¶*) | 0/9 | 2/29 | 1.000 |
|  | 🡹CD8+CD45RA+CCR7- late effector T cells (%*¶*) | 3/9 | 5/29 | 0.232 |
| *Altered other cell subsets* | 🡻CD56+CD16+CD3- natural killer cells (cell/ul) | 1/9 | 21/29 | **0.002*  ***0.298*  ****0.149* |
|  | 🡹CD56+CD16+CD3- natural killer cells (cell/ul) | 1/9 | 0/29 | 0.316 |
|  | 🡻CD56+CD16+CD3- natural killer cells (%‡) | 0/9 | 2/29 | 1.000 |
|  | 🡹CD56+CD16+CD3- natural killer cells (%‡) | 3/9 | 3/29 | 0.357 |
|  | 🡻TCRαβ+CD3+CD4-CD8- double negative T cells (%††) | 0/7 | 2/29 | 1.000 |
|  | 🡹TCRαβ+CD3+CD4-CD8- double negative T cells (%††) | 0/7 | 2/29 | 1.000 |
|  | 🡻CD3+γ+δ+ (%‡) | 0/8 | 2/29 | 1.000 |
|  | 🡹CD3+γ+δ+ (%‡) | 0/8 | 2/29 | 1.000 |
| *Altered CD19+ B-*  *cell subsets* | 🡻CD19+ PAN-B cells (cell/ul) | 4/9 | 20/29 | 0.296 |
|  | 🡹CD19+ PAN-B cells (cell/ul) | 2/9 | 6/29 | 0.689 |
|  | 🡻CD19+ PAN-B cells (%‡) | 4/9 | 14/29 | 0.734 |
|  | 🡹CD19+ PAN-B cells (%‡) | 4/9 | 7/29 | 0.272 |
|  | 🡻CD19+IgD+CD27- naïve B cells (%‡‡) | 1/9 | 3/26 | 1.000 |
|  | 🡹CD19+IgD+CD27- naïve B cells (%‡‡) | 6/9 | 18/26 | 0.709 |
|  | 🡻CD19+IgM++CD38++ transitional B cells (%‡‡) | 4/8 | 6/24 | 0.703 |
|  | 🡹CD19+IgM++CD38++ transitional B cells (%‡‡) | 3/8 | 10/24 | 1.000 |
|  | 🡻CD19+IgD+CD27+ memory B cells (%‡‡) | 5/9 | 13/26 | 0.725 |
|  | 🡹CD19+IgD+CD27+ memory B cells (%‡‡) | 3/9 | 5/26 | 1.000 |
|  | 🡻CD19+IgD-CD27+ switched memory B cells (%‡‡) | 7/9 | 19/26 | 0.550 |
|  | 🡹CD19+IgD-CD27+ switched memory B cells (%‡‡) | 2/9 | 0/26 | 0.111 |
|  | 🡻CD19+CD21+lCD38- CD21low B cells (%‡‡) | 1/8 | 1/25 | 1.000 |
|  | 🡹CD19+CD21+lCD38- CD21low B cells (%‡‡) | 4/8 | 16/25 | 0.714 |
|  | 🡻CD19+IgM-+CD38++ plasmablasts (%‡‡) | 8/10 | 14/24 | 0.106 |
|  | 🡹CD19+IgM-+CD38++ plasmablasts (%‡‡) | 1/10 | 1/24 | 1.000 |
| *Altered*  *immunoglobulin*  *levels* | 🡻IgG (mg/dl)§§ | 4/9 | 21/29 | 0.714 |
|  | 🡹IgG (mg/dl)§§ | 0/9 | 2/29 | 1.000 |
|  | 🡻IgA (mg/dl)§§ | 5/9 | 21/29 | 1.000 |
|  | 🡹IgA (mg/dl)§§ | 0/9 | 3/29 | 0.538 |
|  | 🡻IgM (mg/dl)§§ | 1/9 | 21/29 | **0.002*  ***0.298*  ****0.149* |
|  | 🡹IgM (mg/dl)§§ | 3/9 | 3/29 | 0.357 |
|  | 🡹IgE (mg/dl)§§ | 1/8 | 3/27 | 1.000 |
|  | 🡻IgG1 (mg/dl)§§ | 4/4 | 14/19 | 0.549 |
|  | 🡹IgG1 (mg/dl)§§ | 0/4 | 0/19 | 1.000 |
|  | 🡻IgG2 (mg/dl)§§ | 1/4 | 10/19 | 0.635 |
|  | 🡹IgG2 (mg/dl)§§ | 0/4 | 0/19 | 1.000 |
|  | 🡻IgG3 (mg/dl)§§ | 2/4 | 6/19 | 1.000 |
|  | 🡹IgG3 (mg/dl)§§ | 0/4 | 1/19 | 1.000 |
|  | 🡻IgG4 (mg/dl)§§ | 2/4 | 14/19 | 0.553 |
|  | 🡹IgG4 (mg/dl)§§ | 0/4 | 0/19 | 1.000 |

**Table 5S.** *Abbreviations: WBC, white blood cells.*

** Statistically significant*

*** Bonferroni correction*

**** P-value adjusted as per Benjamini-Hochberg method*

*† % total WBC*

*‡ % total lymphocytes*

*§ % total CD4+ cells ¶ % total CD8+ cells*

*†† % TCRαβ+CD3+ cells*

*‡‡ % total CD19+ cells*

*§§ SI conversion factor: To convert IgG/IgA/IgM to g/L, multiply values by 10²*
