# Supplementary material for: Lymphoproliferation in inborn errors of immunity: From challenging diagnosis to histologic revision
Source: J Hum Immun. 2026 Feb 13;2(2):e20250174. doi: 10.70962/jhi.20250174 (PMC13177383; doi:10.70962/jhi.20250174)
Supplement: Table S6 — shows the salient clues for pathologists in the suspicion of an underlying IEI. [file jhi_20250174_tables6.docx]

**Table S6.** Salient clues for pathologists in the suspicion of an underlying inborn error of immunity

| Histopathological feature | \|  \| \| --- \|  \| **Frequency (n/n)** \| \| --- \| | \|  \| \| --- \|  \| **Key note** \| \| --- \| |
| --- | --- | --- | --- | --- | --- | --- |
| Increased CD8+ T cells within germinal centers (GC) * | 25% (4/16) | Abnormal for GC (normal: very few/none); consider IEI when prominent. |
| Aggregates (>3) of CD303/CD123‑positive plasmacytoid dendritic cells (pDCs) ** | 62.5% (10/16) | pDC aggregates may also be seen in autoimmune conditions; interpret in clinical context. |
| Interfollicular CD4:CD8 ratio skewed toward CD8+ T cells | 87.5% (14/16) | Marked interfollicular CD8 predominance is a frequent finding. |
| Interfollicular PD‑1 expression | 100% (16/16) | Variable intensity; reflects T‑cell activation/exhaustion—supportive but not specific. |
| PTCG‑like features in CVID/CVID-like IEI | 25% (2/8) | Observed in a subset of common‑variable immunodeficiency (CVID) or CVID‑like cases. |
| Increased IRF4‑positive plasma cells within GC § | 37.5% (3/8) | Normally limited to a few plasma cells at the GC periphery; prominence supports CVID/CVID‑like IEI. |

*Table 6S. Legend:*

*° These features are suggestive but not pathognomonic for inborn errors of immunity (IEI); similar changes can occur in non‑IEI reactive lymph nodes that exhibit immune dysregulation.*

** Normal pattern: very few or absent CD8+ T cells within germinal centers.*

*** pDC aggregates are also frequently observed in autoimmune diseases; their presence should prompt correlation with clinical and laboratory data.*

*§ Normal distribution of IRF4+ plasma cells in germinal centers: a few cells located at the periphery, representing exiting plasmablasts.*
